# Supplementary material for: Engineering electronic structure to prolong relaxation times in molecular qubits by minimising orbital angular momentum
Source: Nat Commun. 2019 Jul 26;10:3330. doi: 10.1038/s41467-019-11309-3 (PMC6659626; doi:10.1038/s41467-019-11309-3)
Supplement: Supplementary file 1 — Supplementary Information [file 41467_2019_11309_MOESM1_ESM.pdf]

Electronic Supplementary Information

Engineering electronic structure to prolong relaxation times in molecular qubits by minimising orbital angular momentum

Ana-Maria Ariciu,[a,b,§] David H. Woen,[c,§] Daniel N. Huh,[c] Lydia Nodarki,[a] Andreas K. Kostopoulos,[a] Conrad A. P. Goodwin,[a] Nicholas F. Chilton,[a] Eric J. L. McInnes,[a,b] Richard E. P. Winpenny,[a,*] William J. Evans,[c,*] and Floriana Tuna[a,b,*]

[a]*School of Chemistry, The University of Manchester, Oxford Road, Manchester, M13 9PL, UK.*

[b]*Photon Science Institute, The University of Manchester, Oxford Road, Manchester, M13 9PL, UK.*

[c]*Department of Chemistry, University of California, Irvine, California, 92697-2025, United States.*

§ These authors contributed equally.

Supplementary Figures

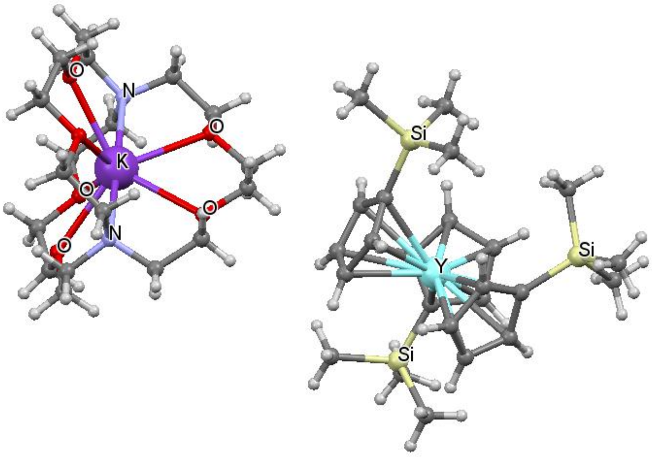

Supplementary Figure 1. Molecular structure of **1**: light blue, Y; yellow, Si; purple, K; light purple, N; red, O; grey, C; light grey, H.[2]

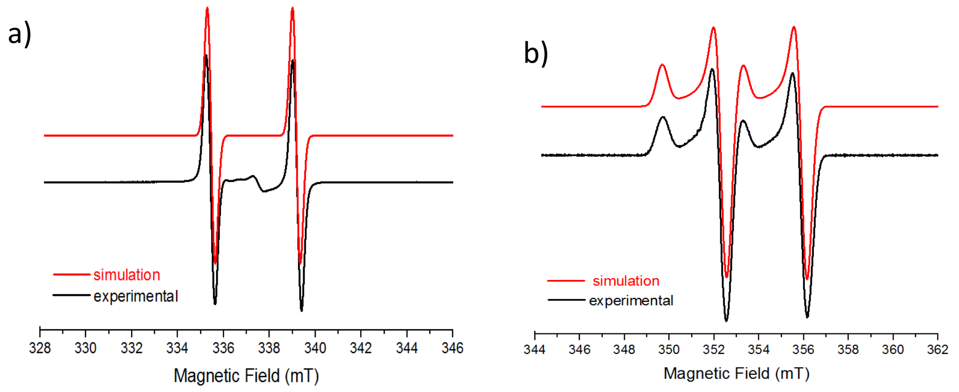

Supplementary Figure 2. Experimental (black) and simulated (red) X-band CW-EPR spectra for **1** (10 mM in THF). Simulation parameters: g_{iso} = 1.991; $A_{iso}(^{89}Y)$ = 102 MHz: a) 295 K fluid solution at 9.40 GHz; b) 50 K frozen solution at 9.85 GHz.

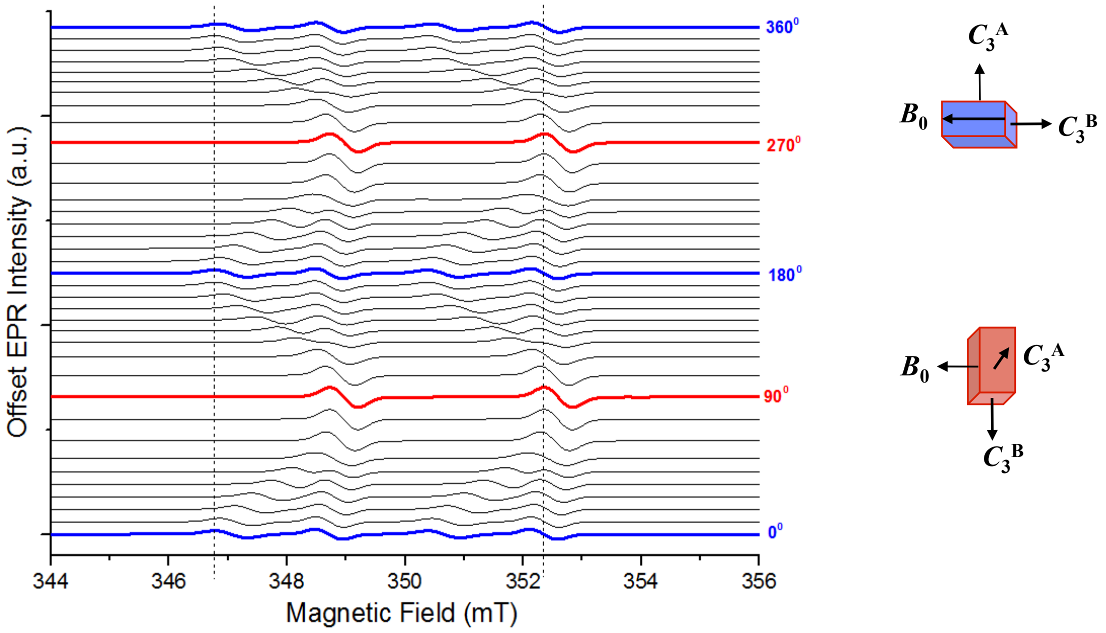

Supplementary Figure 3. X-band CW EPR road-map for a single crystal of **~2% 1@5** at 200 K and 9.755 GHz, following stepwise rotation around C_3 of molecules **A** (see above). At positions highlighted in blue, B_0 is along the C_3 axis of molecules **B**, and nearly perpendicular to C_3 of molecules **A**. At positions highlighted in red, B_0 is perpendicular to C_3 of both **A** and **B**, and thus the measured signal allows determination of $g_{x,y}$. The effective g-values are: g_z = 1.999 and $g_{x,y}$ = 1.986.

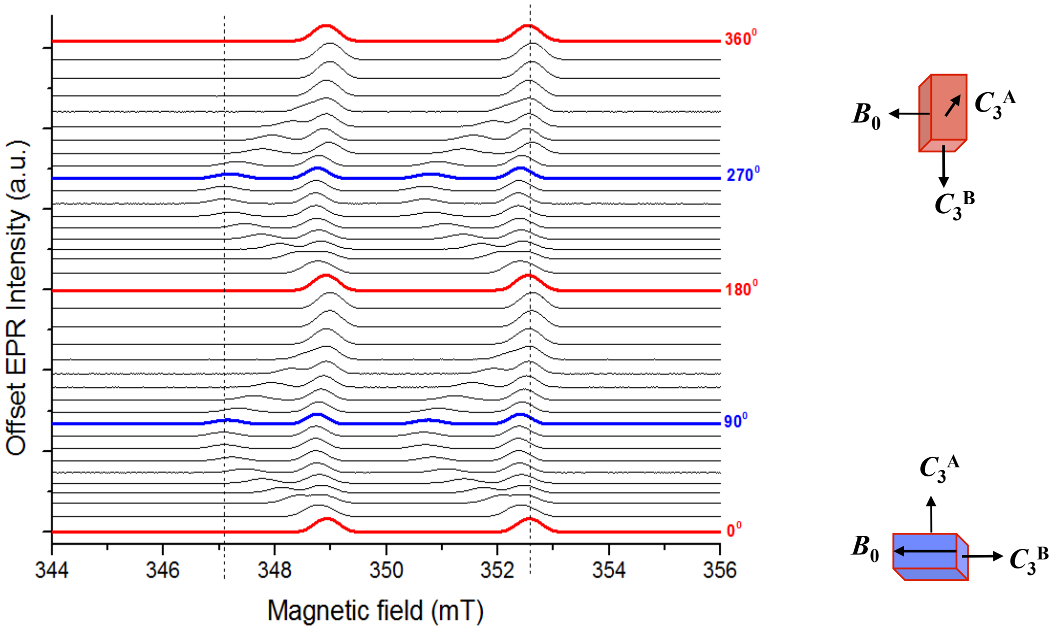

Supplementary Figure 4. EDFS road-map (angular dependence profile) for single crystal **~2% 1@5** following stepwise rotation **(**10 deg. increments) around C_3 of molecules **A,** recorded at 200 K and 9.755 GHz (X-band). At positions highlighted in red, B_0 is perpendicular to C_3 of molecules **A** and **B;** the observed double resonance EPR signal is simulated with $g_{x,y}$ = 1.986, and $A_{x,y}$ (^{89}Y) = 101.3 MHz. At positions highlighted in blue, B_0 is along the C_3 axis of molecules **B**, and nearly perpendicular to C_3 of molecules **A**. Simulation of the data provides g_z = 1.999, $g_{x,y}$ = 1.986, A_z = 99.5 and $A_{x,y}$ = 101.3 MHz (see Fig. 2b and Table 2 in the main text).

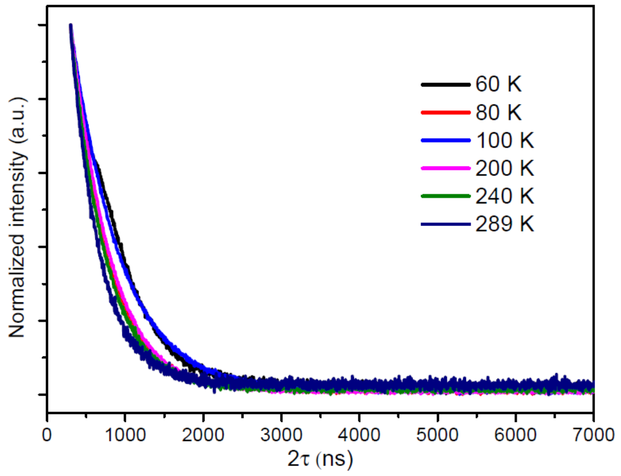

Supplementary Figure 5. Normalized Hahn echo signal intensities as a function of the inter-pulse delay 2τ, for **~2% 1@5** (single crystal) at B_0 = 349 mT (**OP2** in Fig. 2b; $B_0 \perp C_3$) and selected temperatures.

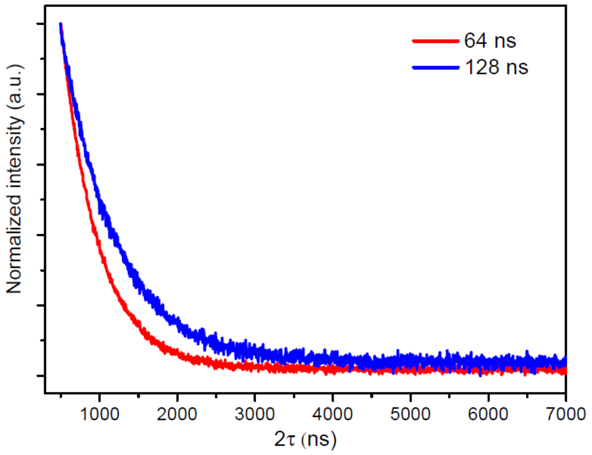

Supplementary Figure 6. Normalized Hahn echo signal intensities as a function of the inter-pulse delay 2τ for **~2% 1@5** (single crystal) at B_0 = 352.6 mT (**OP4** in Figure 2b; $B_0 \perp C_3$) and 100 K. The rate of relaxation varies with the pulse length, with more selective (longer) pulses being more effective in suppressing proton modulations.

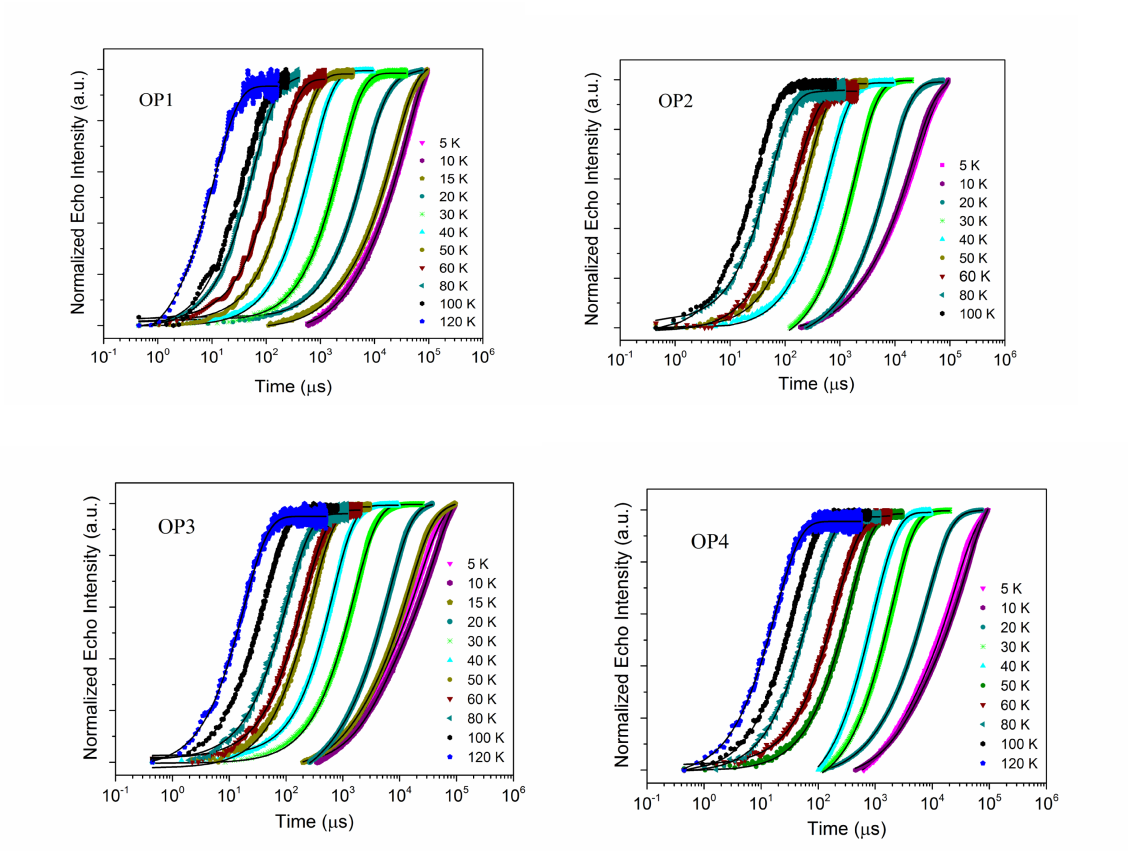

Supplementary Figure 7. Inversion recovery data for **1** (10 mM; THF) at selected temperatures between 5 and 120 K, and at B_0 of 349.8 mT (**OP1**), 350.3 mT (**OP2**), 352.2 mT (**OP3**), and 355.9 mT (**OP4**). The red lines are best fits to the biexponential model (Eq. 3), giving the parameters in Supplementary Table 3. The observer positions OP1-OP4 are indicated in Fig. 2a (main text).

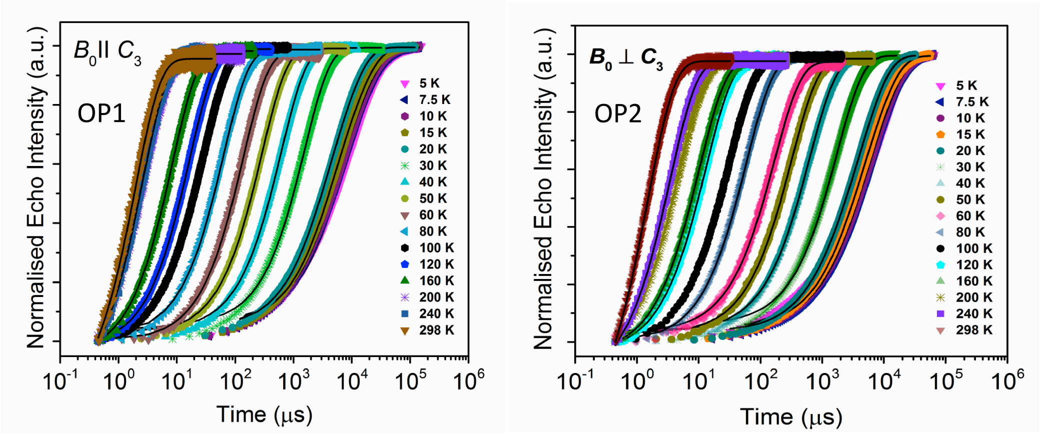

Supplementary Figure 8. Inversion recovery data for **~2% 1@5** (single crystal) at different temperatures between 5 and 298 K, and at B_0 of 347 mT (**OP1**), or 349 mT (**OP2**). The solid lines are best fits to the biexponential model (Eq. 3), giving the parameters in Supplementary Table 4. The observer positions OP1-OP4 are indicated in Fig. 2b (main text).

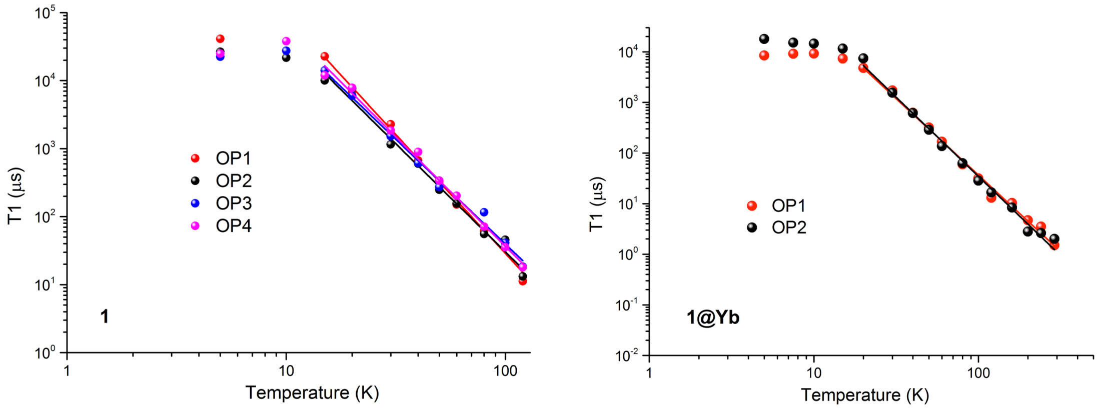

Supplementary Figure 9. Temperature dependence of T_1 for (**left**) **1** (THF) and (**right**) **~2% 1@5** (single crystal), at X-band and different observable positions, indicated in Figs. 2a and 2b. The solid lines represent the best fits to equation $T_1^{-1} = CT^n$ with n = 3 - 3.5.

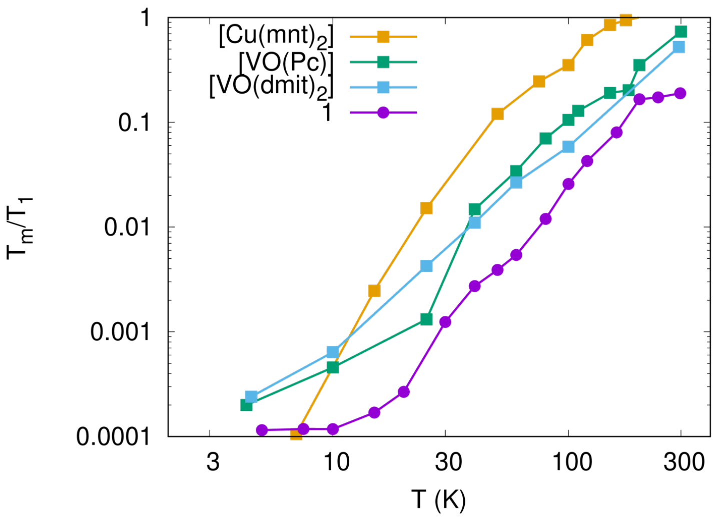

Supplementary Figure 10. T_m/T_1 as a function of temperature for: ~2% **1@5**; [Cu(mnt)$_2$] (0.001% solid-state dilution) (8), [VO(Pc)] (0.001% solid-state dilution) (26); [VO(dmit)$_2$] (5% solid-state dilution) (11).

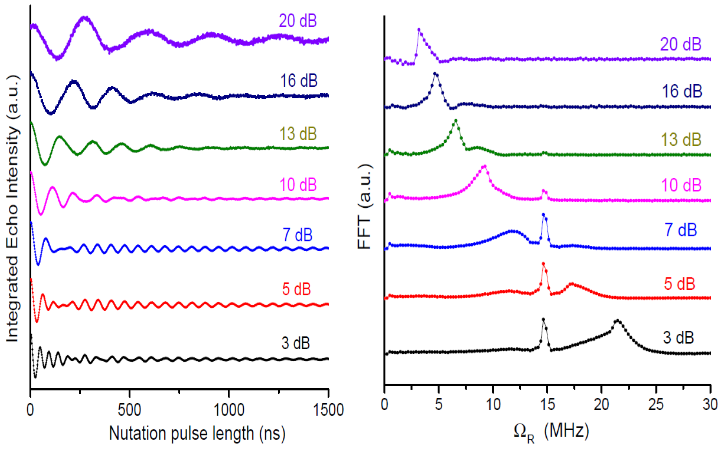

Supplementary Figure 11. (Left) Rabi oscillation for **1** (THF) at 120 K and B_0 = 349.8 mT (**OP1**; Fig. 2a), acquired with different microwave attenuations; (Right) corresponding Fourier transforms.

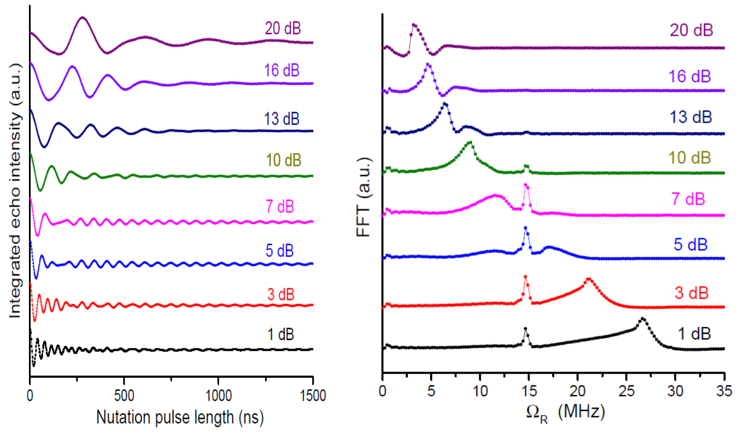

Supplementary Figure 12. (Left) Rabi oscillations for **1** (THF) at 40 K and B_0 = 349.8 mT (**OP1**; Fig. 2a), acquired with different microwave attenuations; (Right) corresponding Fourier transforms.

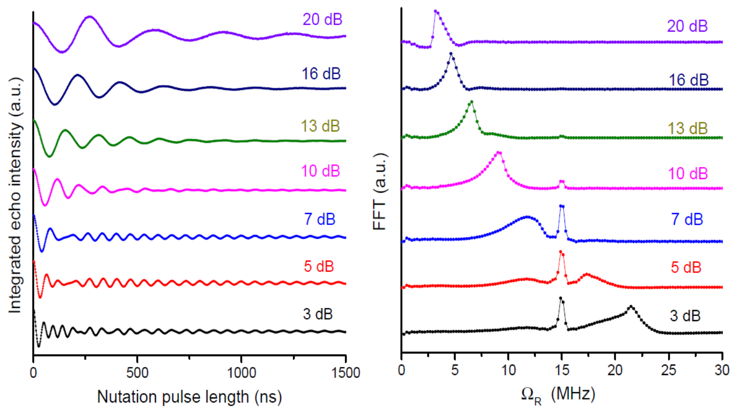

Supplementary Figure 13. (Left) Rabi oscillation for **1** (THF) at 120 K and B_0 = 355.9 mT (**OP4**; Fig. 2a), acquired with different microwave attenuations; (Right) corresponding Fourier transforms.

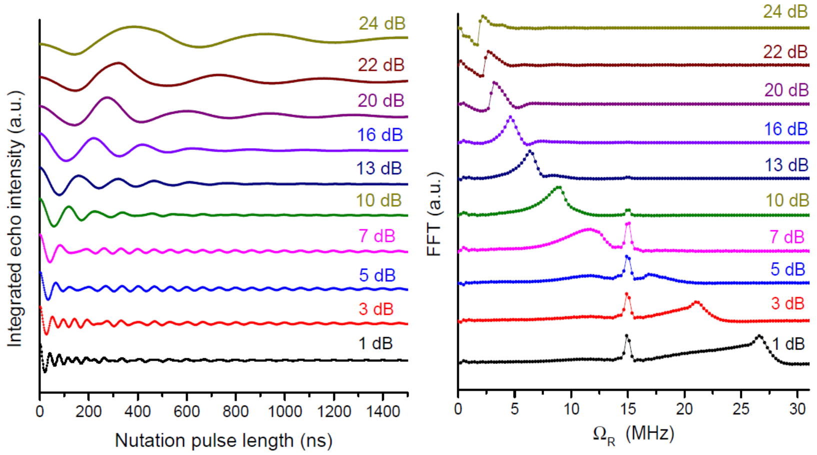

Supplementary Figure 14. (Left) Rabi oscillation for **1** (THF) at 40 K and B_0 = 355.9 mT (**OP4**; Fig. 2a), acquired with different microwave attenuations; (Right) corresponding Fourier transforms.

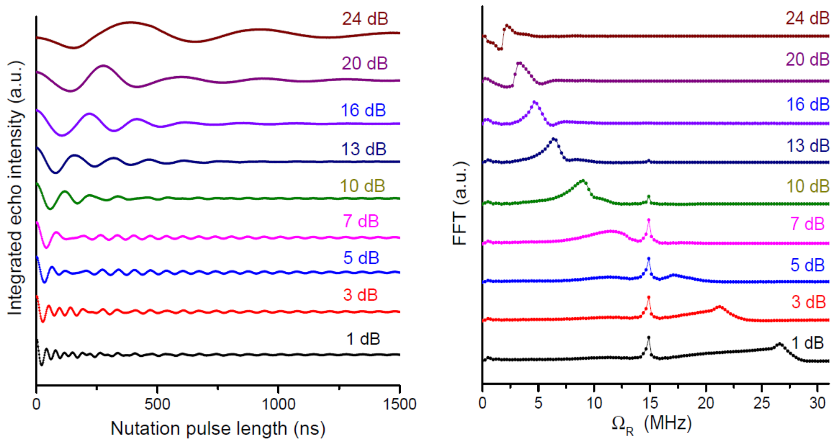

Supplementary Figure 15. (Left) Rabi oscillation for **1** (THF) at 40 K and B_0 = 352.2 mT (**OP3**; Fig. 2a), acquired with different microwave attenuations; (Right) corresponding Fourier transforms.

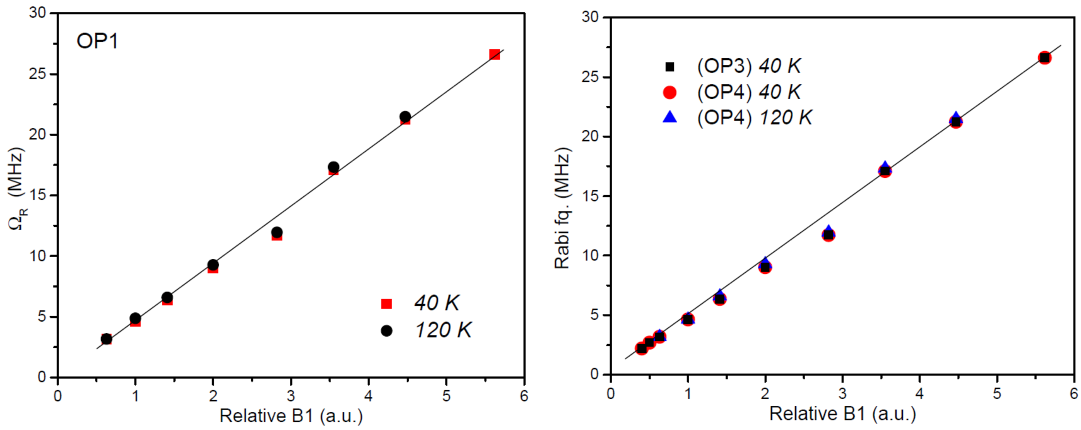

Supplementary Figure 16. B_1 dependence of the Rabi frequency (Ω_R) for **1** (THF) measured at 40 and 120 K, for different observable positions (Fig. 2a). The solid line is a guide for the eye emphasizing the linear behaviour ($B_1 \propto \sqrt{P}$, where P is the microwave power).

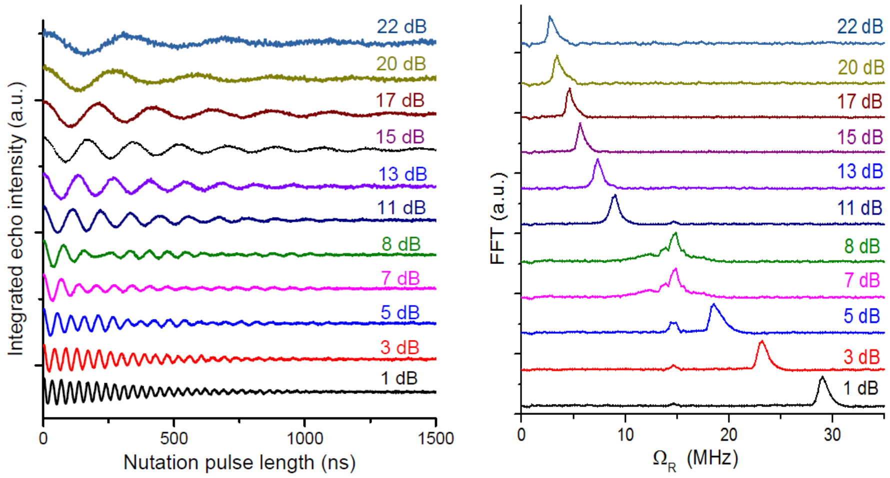

Supplementary Figure 17. (Left) Rabi oscillation for **~2% 1@5** (single crystal) at 298 K and B_0 **=** 347 mT (**OP1**; Fig. 2b; $B_0||C_3$), acquired with different microwave attenuations; (Right) corresponding Fourier transforms.

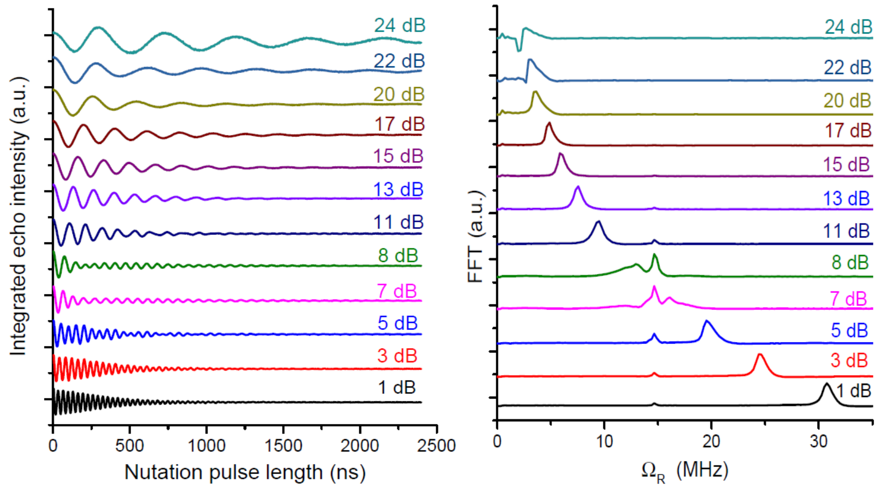

Supplementary Figure 18. (Left) Rabi oscillation for **~2% 1@5** (single crystal) at 120 K and B_0 = 347 mT (**OP1;** Fig 2b; $B_0 \parallel C_3$), acquired with different microwave attenuations; (Right) corresponding Fourier transforms.

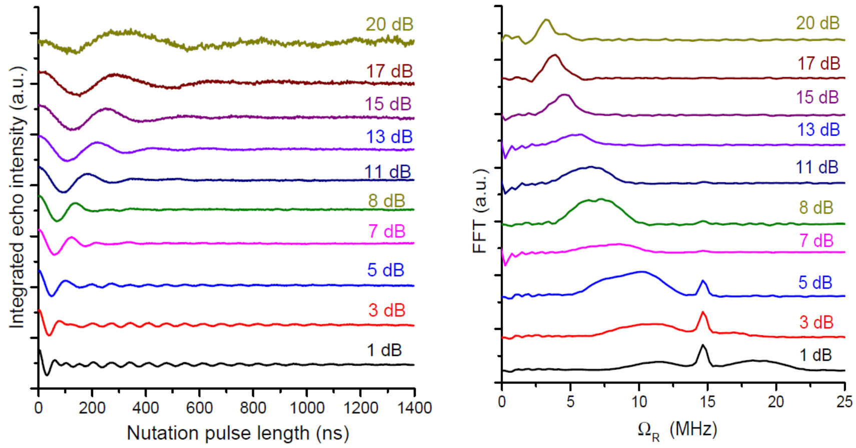

Supplementary Figure 19. (Left) Rabi oscillation for **~2% 1@5** (single crystal) at 298 K and B_0 = 349 mT (**OP2;** Fig. 2b; $B_0 \perp C_3$**)**, acquired with different microwave attenuations; (Right) corresponding Fourier transforms.

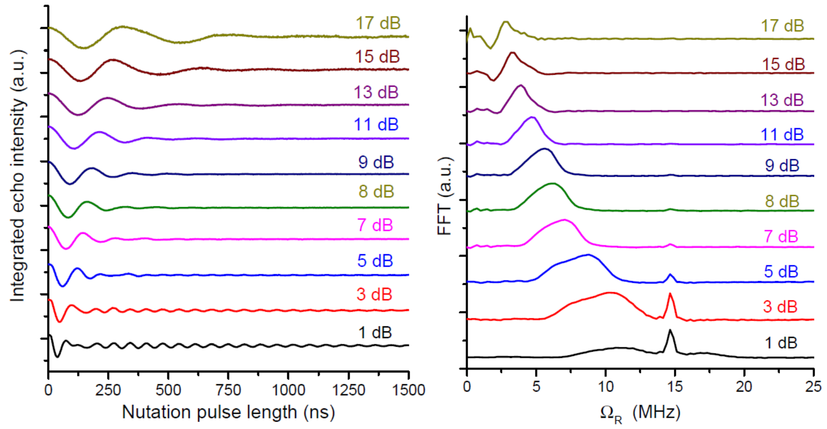

Supplementary Figure 20. (Left) Rabi oscillation for **~2% 1@5** (single crystal) at 120 K and B_0 = 349 mT (**OP2;** Fig. 2b; $B_0 \perp C_3$) acquired with different microwave attenuations; (Right) corresponding Fourier transforms.

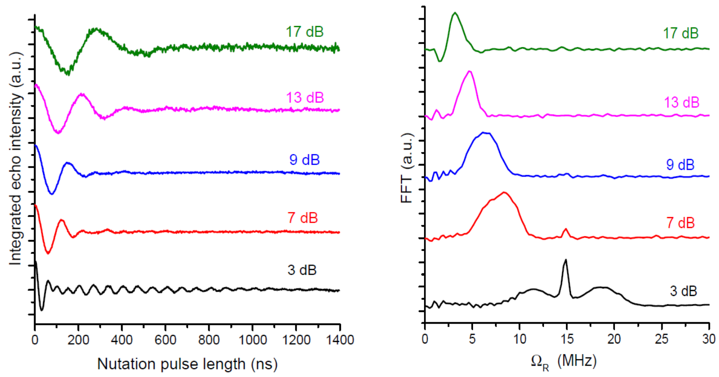

Supplementary Figure 21. (Left) Rabi oscillation for **~2% 1@5** (single crystal) at 298 K and B_0 = 352.7 mT (**OP4;** Fig. 2b; $B_0 \perp C_3$), acquired with different microwave attenuations; (Right) corresponding Fourier transforms.

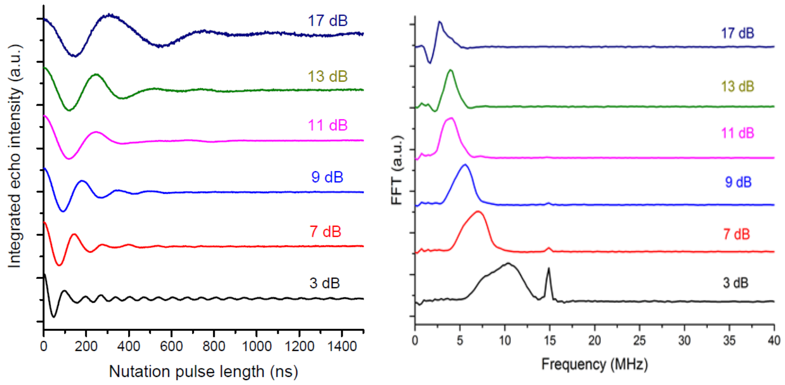

Supplementary Figure 22. (Left) Rabi oscillation for **~2% 1@5** (single crystal) at 120 K and 352.7 mT (**OP4;** Fig. 2b; $B_0 \perp C_3$), acquired with different microwave attenuations; (Right) corresponding Fourier transforms.

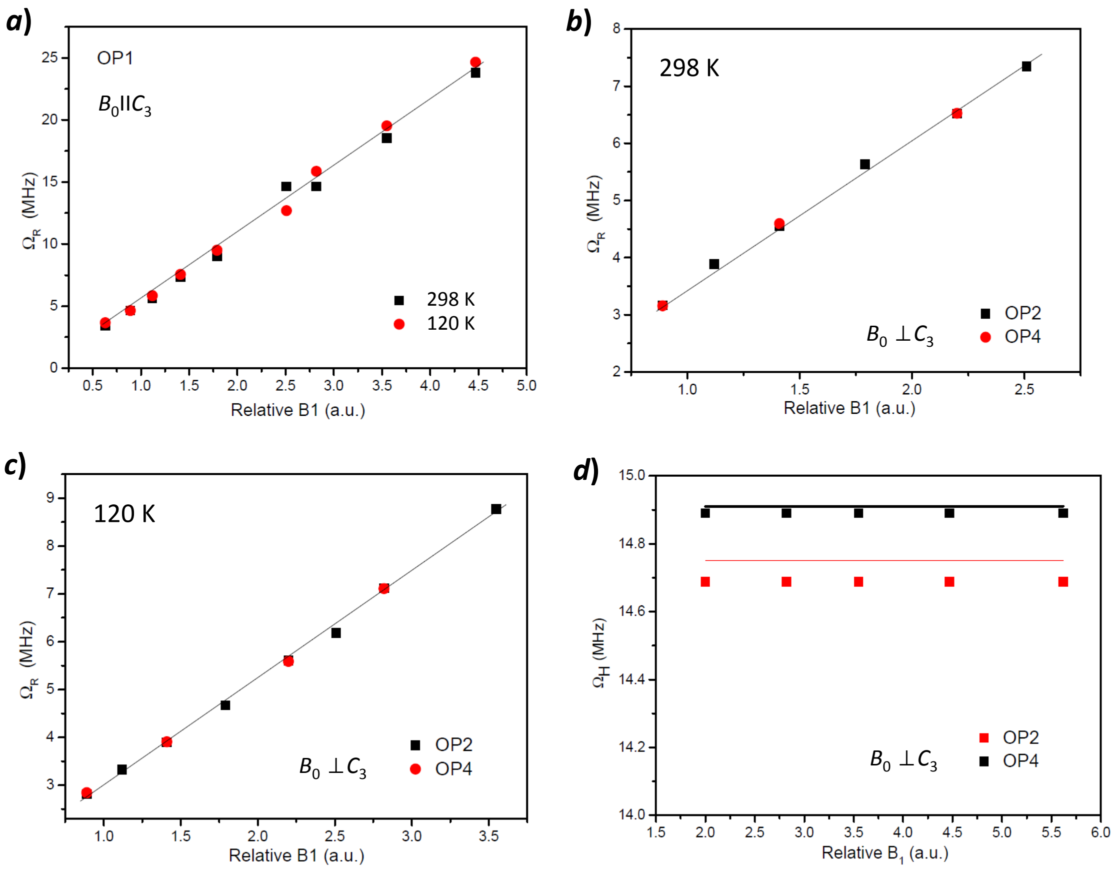

Supplementary Figure 23. B_1 dependence of the (**a-c**) Rabi frequency (Ω_R), and (**d**) ^{1}H nuclear frequency (Ω_H), for **~2% 1@5** (single crystal), measured at different observable positions (Fig. 2b), and temperatures of 120 and 298 K. The solid line is a guide for the eye emphasizing the linear behaviour ($B_1 \propto \sqrt{P}$, where P is the microwave power).

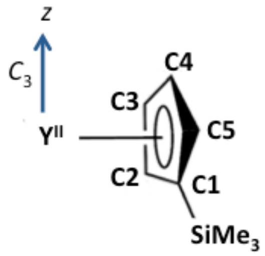

Supplementary Figure 24. Schematic representation for the binding of a Cp' ring in **1,** and the direction of the molecular C_3 axis.

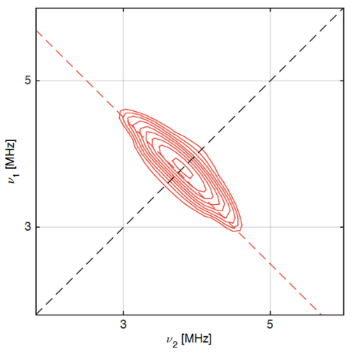

Supplementary Figure 25. Calculation of the ^{13}C HYSCORE spectrum of **1** based on the dipole model only (see text). The dashed-red antidiagonal lines mark the ^{13}C Larmor frequency.

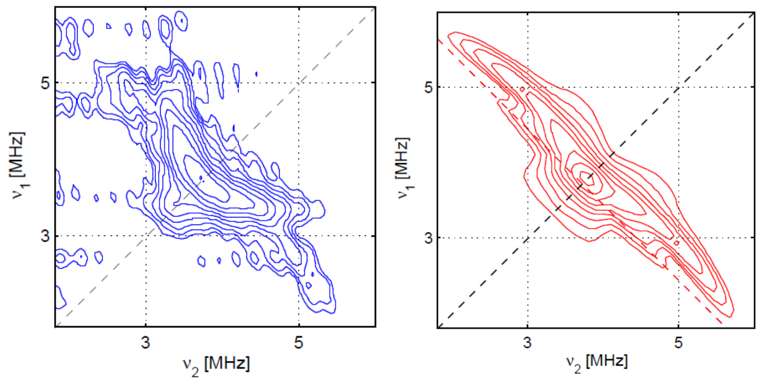

Supplementary Figure 26. (Left) ^{13}C HYSCORE spectrum for **1** (THF) at B_0 = 349.7 mT (**OP1**; Fig. 2a), T = 50 K and X-band (9.848 GHz) (Right) Calculation taking into account the point-dipole interactions and the associated spin densities at $C^{2,5}$ and $C^{3,4}$, yielding: $A_{||}^{C2,5}$ = 2.8 MHz; $A_{\perp}^{C2,5}$ **=** 0.4 MHz, and $A_{||}^{C3,4}$ = 0.825 MHz; and $A_{\perp}^{C3,4}$ **=** 0.3 MHz. The dashed-red antidiagonal line marks the ^{13}C Larmor frequency.

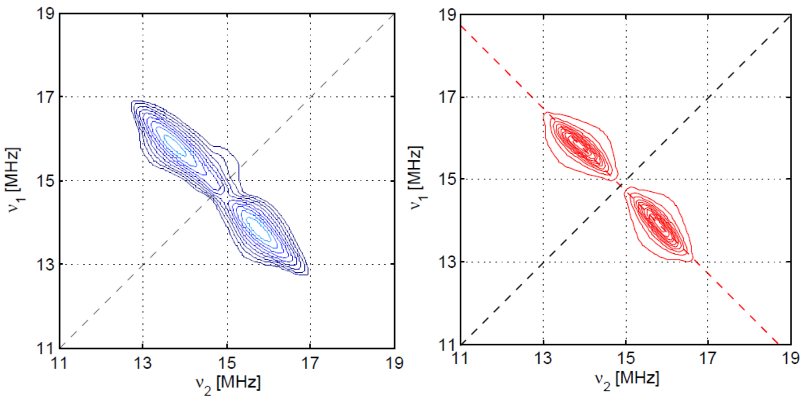

Supplementary Figure 27. (Left) ^{1}H HYSCORE spectrum for **1** (THF) at B_0 = 349.7 mT (**OP1**; Fig. 2a), T = 50 K, and X-band (9.848 GHz). (Right) Calculation based on the model described in the text, with a_{iso} =-0.7 MHz. The dashed-red antidiagonal line marks the ^{1}H Larmor frequency.

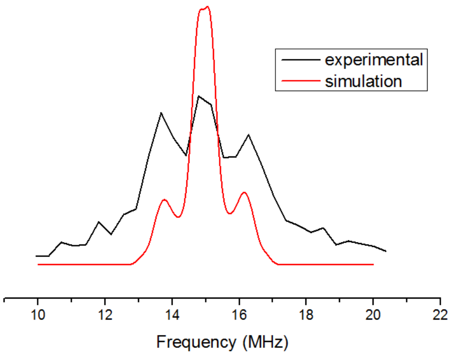

Supplementary Figure 28. [1]H Davies-ENDOR spectrum of **1** (THF) at B_0 = 352.6 mT (**OP3**; Fig. 1b) and T = 50 K (black), and its calculation based on the model described in the text (red).

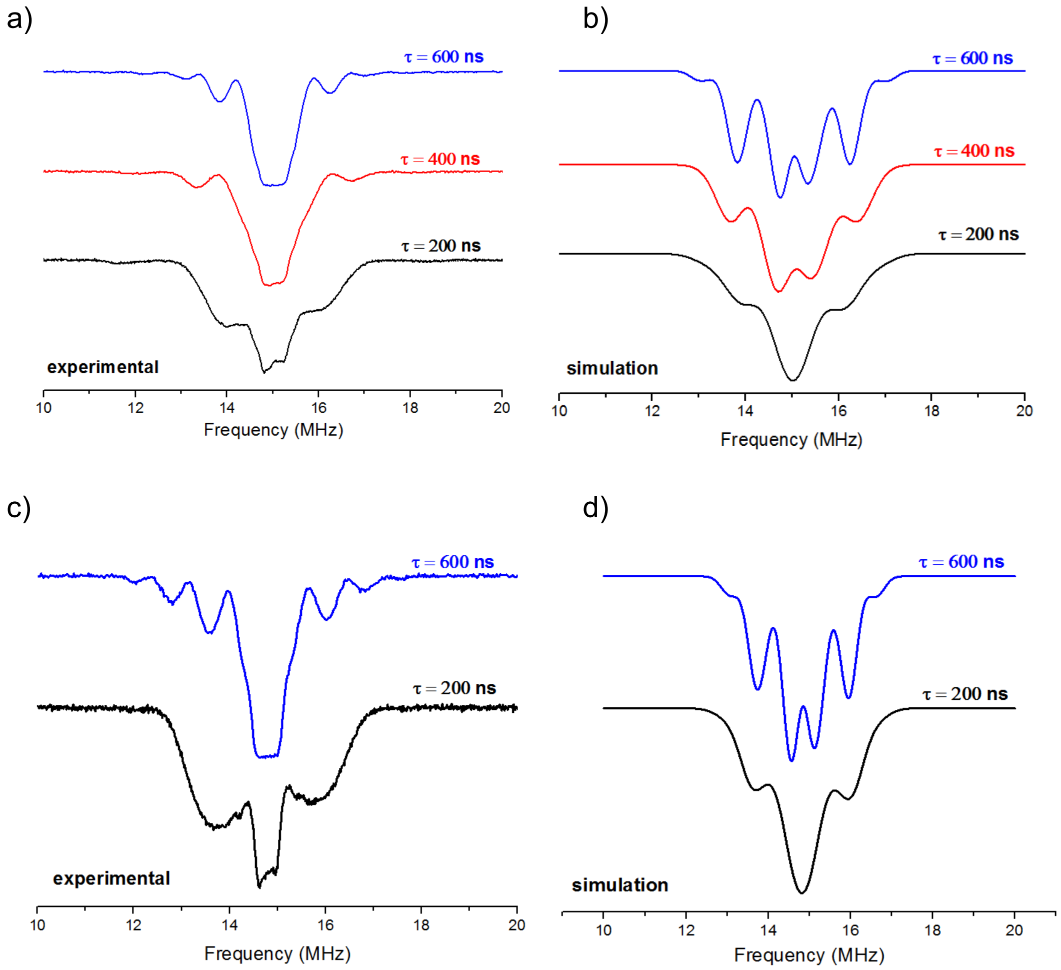

Supplementary Figure 29. ENDOR spectroscopy of **1**. a) [1]H Mims-ENDOR spectra of **1 (THF)** at B_0 = 352.6 mT (**OP3**; Fig. 1b), T = 10 K and different τ values; b) Correspondent calculations based on the model described in the text; c) [1]H Mims-ENDOR spectra at 10 K and B_0 = 349 mT (**OP1**; Fig. 2a); d) Simulation of the data in Supplementary Figure 29c. Simulations used EasySpin.

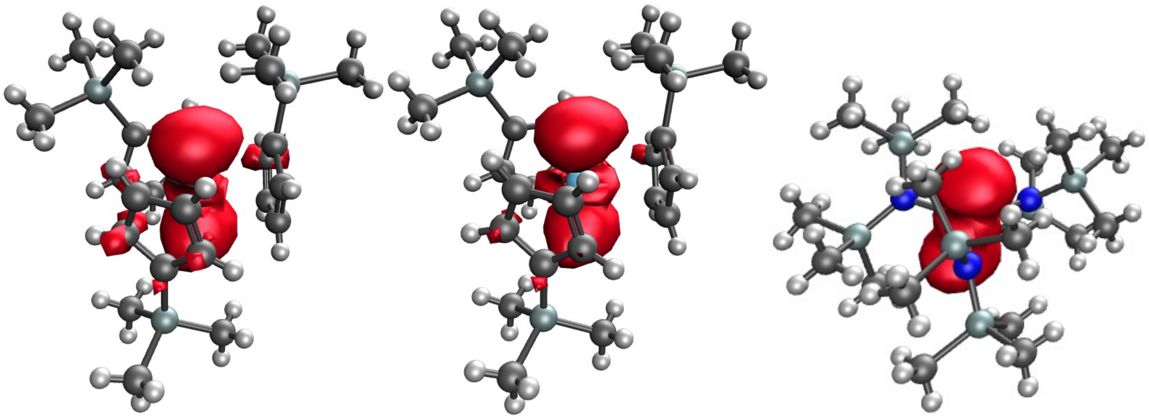

Supplementary Figure 30. A rendering of the spin density from DFT for the crystal structure anions in **2-4** (left to right, respectively).

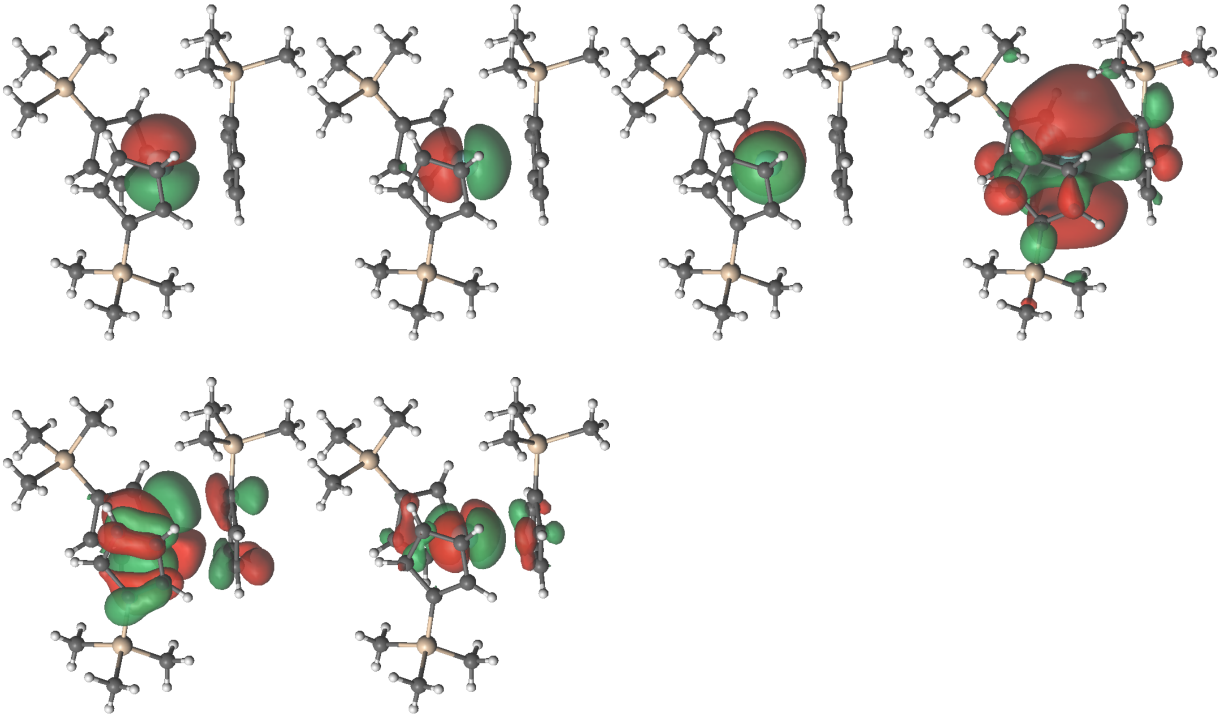

Supplementary Figure 31. Orbitals 130 – 135 in the active space in the CASSCF calculations for the crystalline geometry of **1** (isovalue = 0.02).

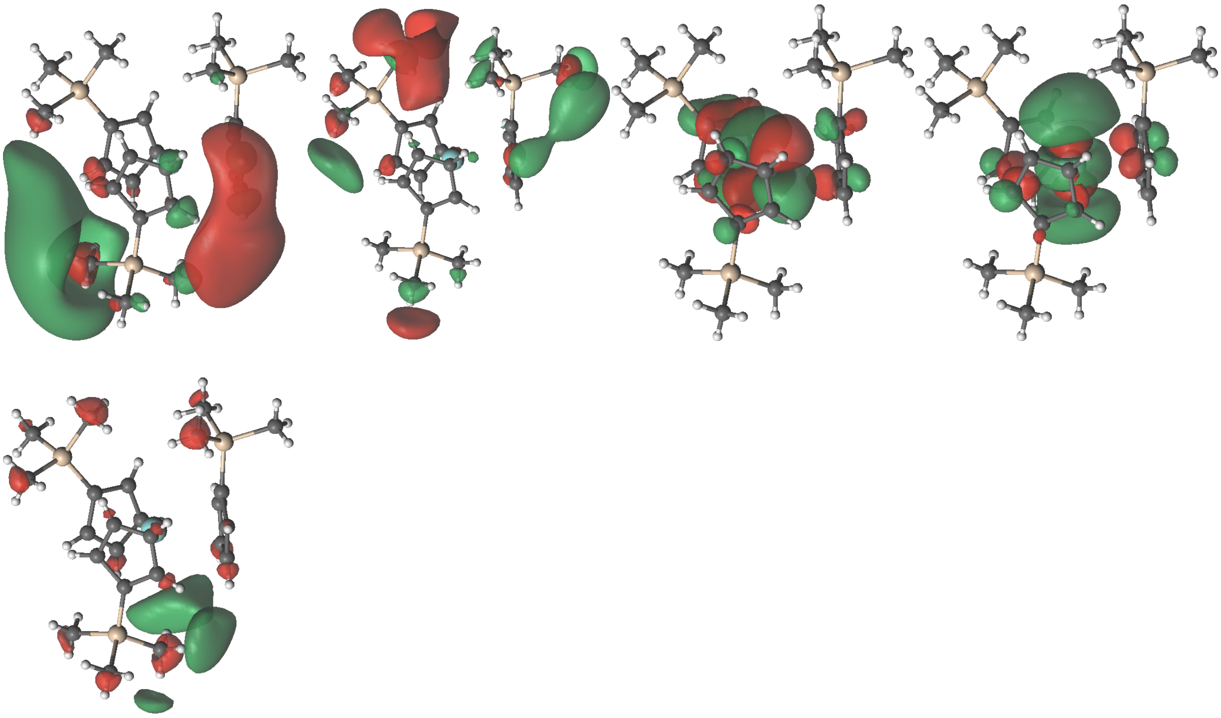

Supplementary Figure 32. Orbitals 136 – 140 in the active space in the CASSCF calculations for the crystalline geometry of **1** (isovalue = 0.02).

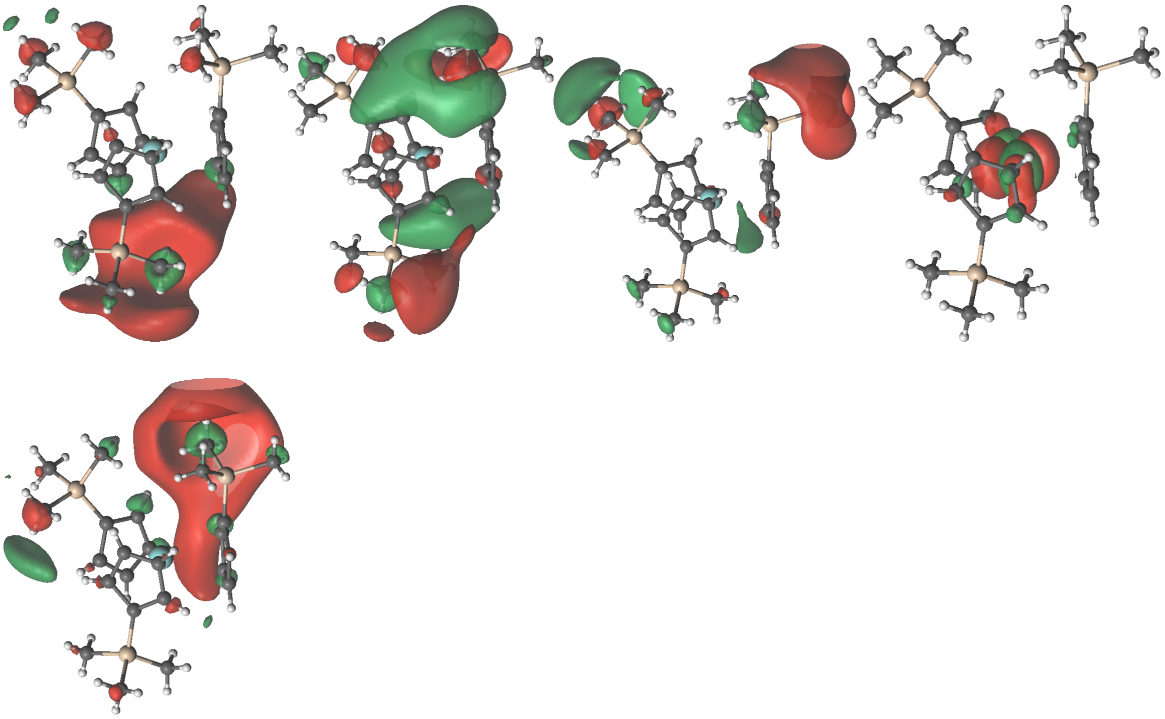

Supplementary Figure 33. Orbitals 141 – 145 in the active space in the CASSCF calculations for the crystalline geometry of **1** (isovalue = 0.02).

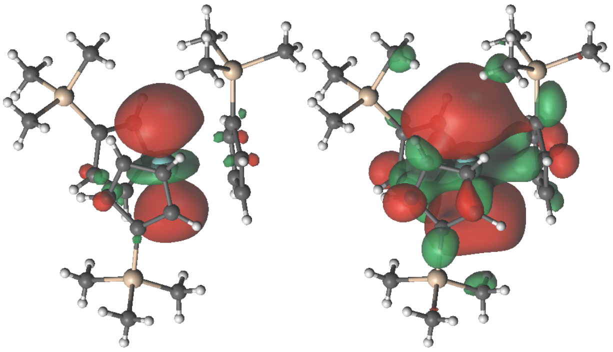

Supplementary Figure 34. Natural SOMO for state 1 in the CASSCF calculations for the crystalline geometry of **1**. Isovalue = 0.04 (left) and 0.02 (right).

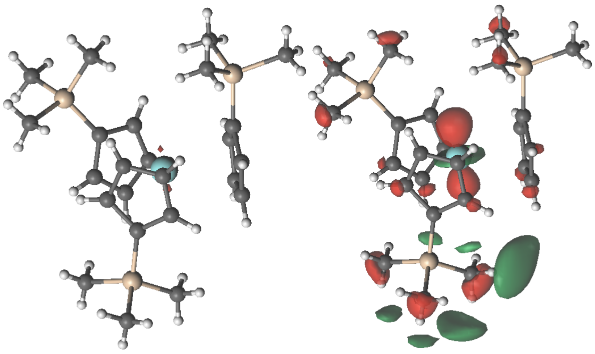

Supplementary Figure 35. Natural SOMO for state 2 in the CASSCF calculations for the crystalline geometry of **1**. Isovalue = 0.04 (left) and 0.02 (right).

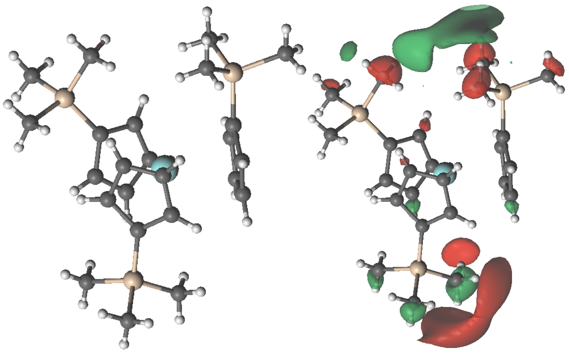

Supplementary Figure 36. Natural SOMO for state 3 in the CASSCF calculations for the crystalline geometry of **1**. Isovalue = 0.04 (left) and 0.02 (right).

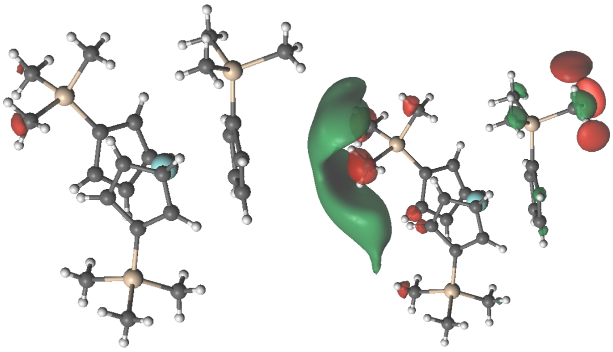

Supplementary Figure 37. Natural SOMO for state 4 in the CASSCF calculations for the crystalline geometry of **1**. Isovalue = 0.04 (left) and 0.02 (right).

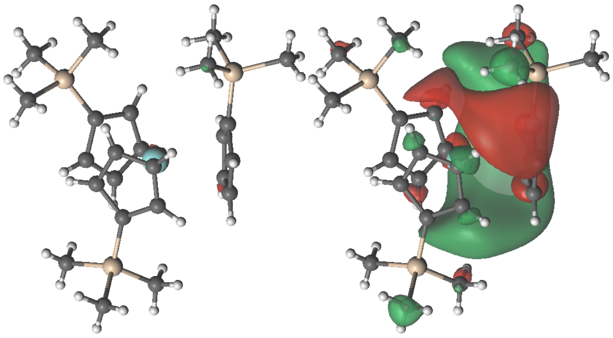

Supplementary Figure 38. Natural SOMO for state 5 in the CASSCF calculations for the crystalline geometry of **1**. Isovalue = 0.04 (left) and 0.02 (right).

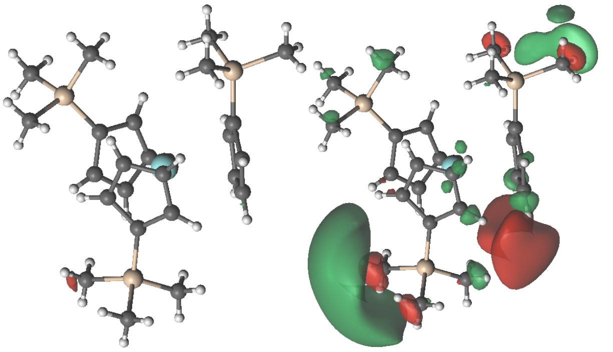

Supplementary Figure 39. Natural SOMO for state 6 in the CASSCF calculations for the crystalline geometry of **1**. Isovalue = 0.04 (left) and 0.02 (right).

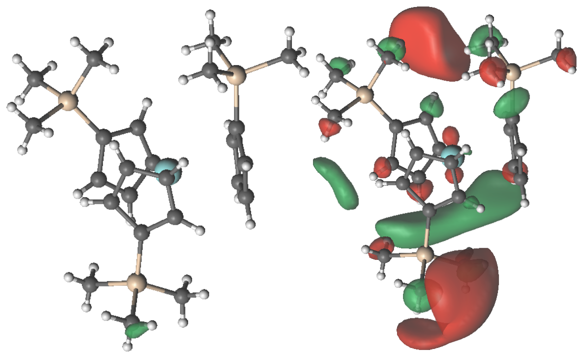

Supplementary Figure 40. Natural SOMO for state 7 in the CASSCF calculations for the crystalline geometry of **1**. Isovalue = 0.04 (left) and 0.02 (right).

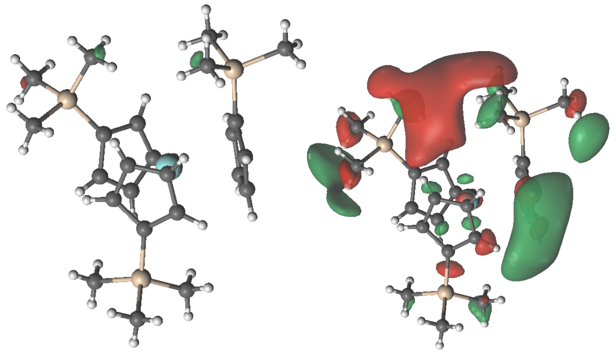

Supplementary Figure 41. Natural SOMO for state 8 in the CASSCF calculations for the crystalline geometry of **1**. Isovalue = 0.04 (left) and 0.02 (right).

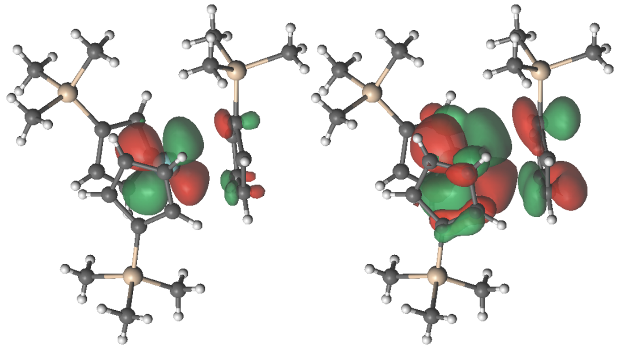

Supplementary Figure 42. Natural SOMO for state 9 in the CASSCF calculations for the crystalline geometry of **1**. Isovalue = 0.04 (left) and 0.02 (right).

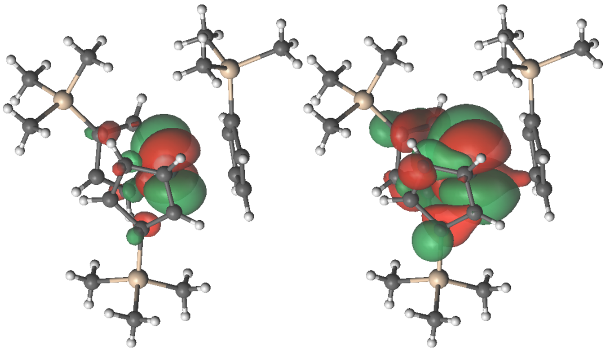

Supplementary Figure 43. Natural SOMO for state 10 in the CASSCF calculations for the crystalline geometry of **1**. Isovalue = 0.04 (left) and 0.02 (right).

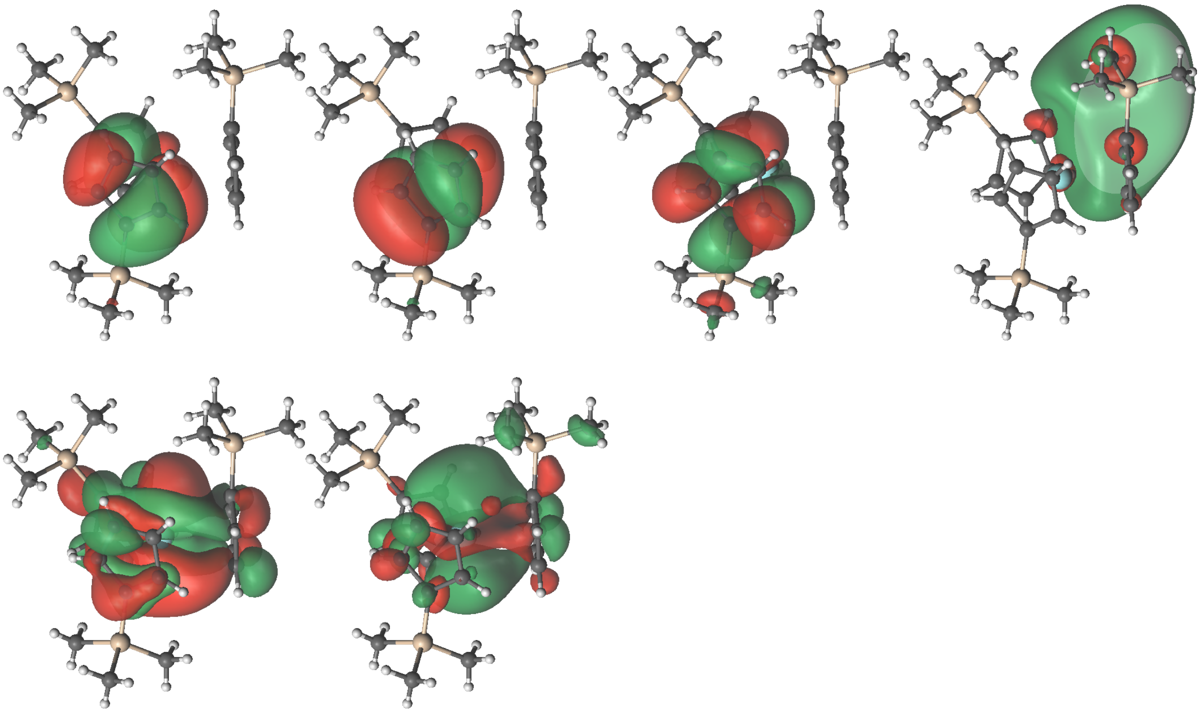

Supplementary Figure 44. Orbitals 131 – 136 in the active space in the CASSCF calculations for the crystalline geometry of **1** with a sphere of point charges (isovalue = 0.02).

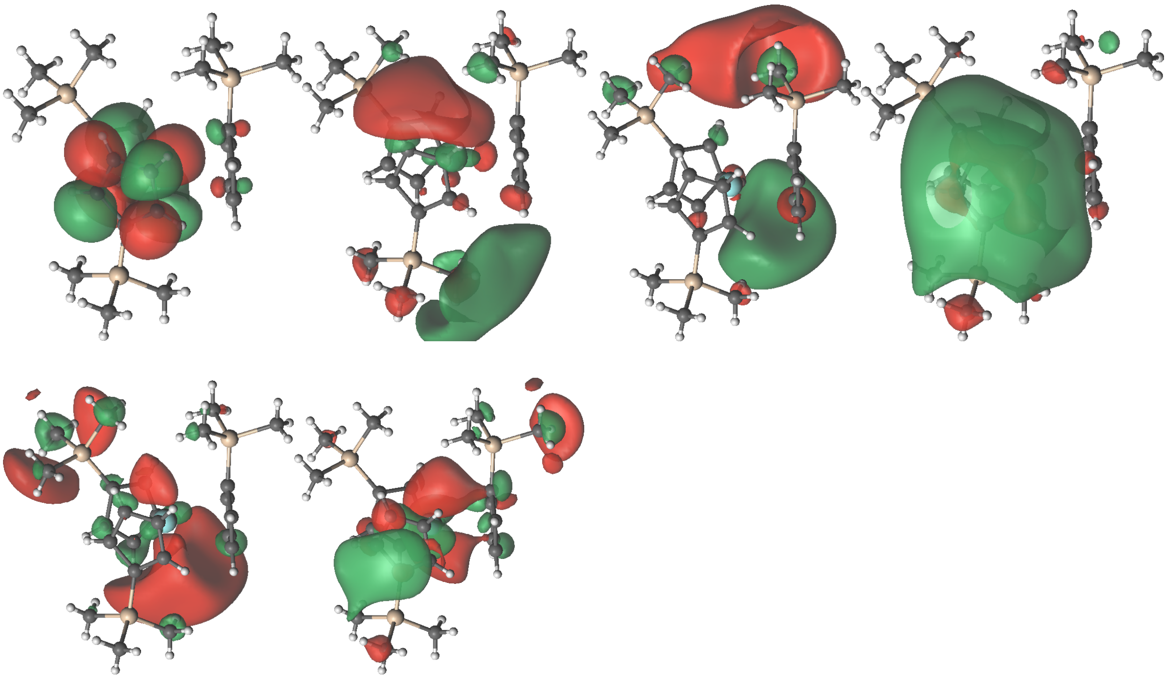

Supplementary Figure 45. Orbitals 137 – 142 in the active space in the CASSCF calculations for the crystalline geometry of **1** with a sphere of point charges (isovalue = 0.02).

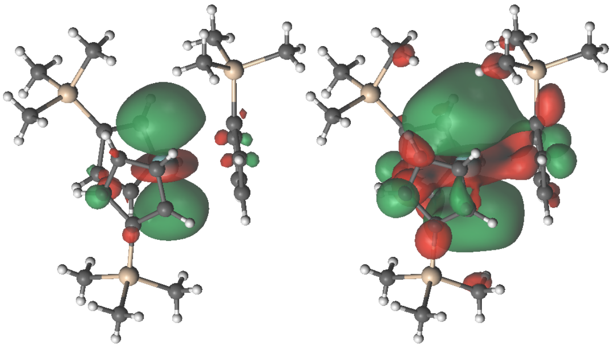

Supplementary Figure 46. Natural SOMO for state 1 in the CASSCF calculations for the crystalline geometry of **1** with a sphere of point charges. Isovalue = 0.04 (left) and 0.02 (right).

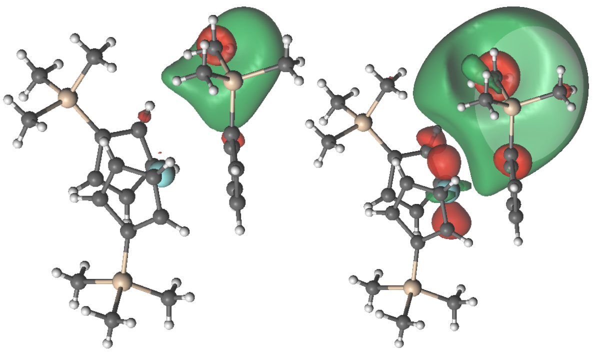

Supplementary Figure 47. Natural SOMO for state 2 in the CASSCF calculations for the crystalline geometry of **1** with a sphere of point charges. Isovalue = 0.04 (left) and 0.02 (right).

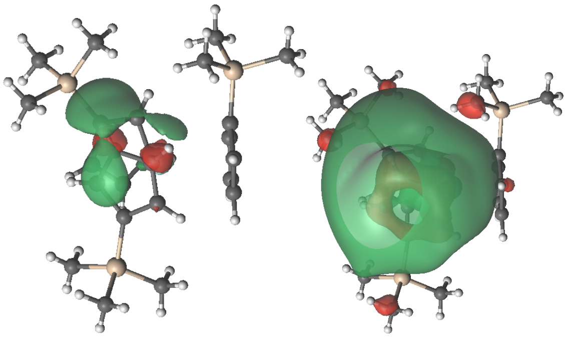

Supplementary Figure 48. Natural SOMO for state 3 in the CASSCF calculations for the crystalline geometry of **1** with a sphere of point charges. Isovalue = 0.04 (left) and 0.02 (right).

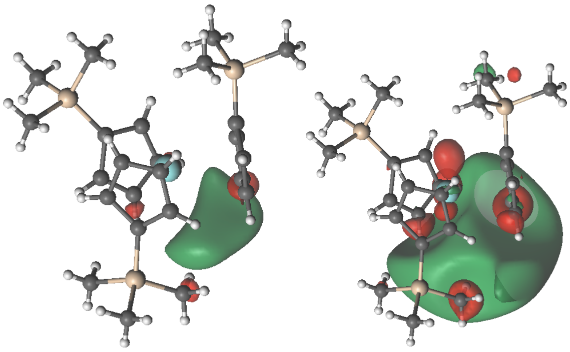

Supplementary Figure 49. Natural SOMO for state 4 in the CASSCF calculations for the crystalline geometry of **1** with a sphere of point charges. Isovalue = 0.04 (left) and 0.02 (right).

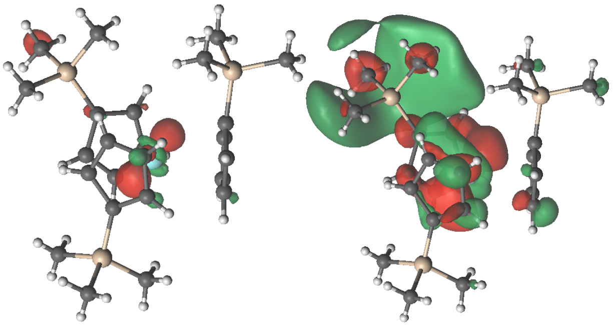

Supplementary Figure 50. Natural SOMO for state 5 in the CASSCF calculations for the crystalline geometry of **1** with a sphere of point charges. Isovalue = 0.04 (left) and 0.02 (right).

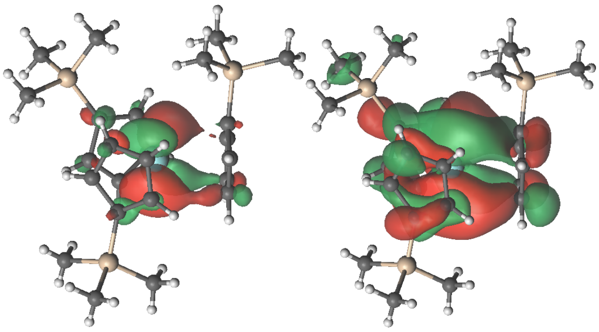

Supplementary Figure 51. Natural SOMO for state 6 in the CASSCF calculations for the crystalline geometry of **1** with a sphere of point charges. Isovalue = 0.04 (left) and 0.02 (right).

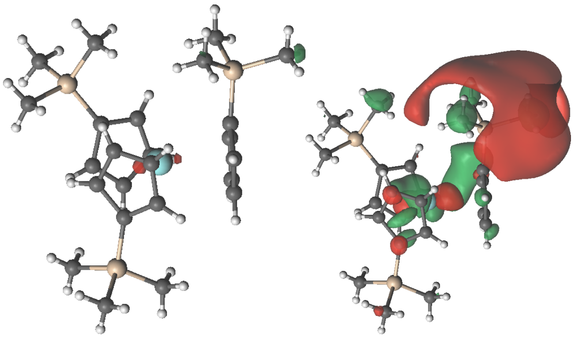

Supplementary Figure 52. Natural SOMO for state 7 in the CASSCF calculations for the crystalline geometry of **1** with a sphere of point charges. Isovalue = 0.04 (left) and 0.02 (right).

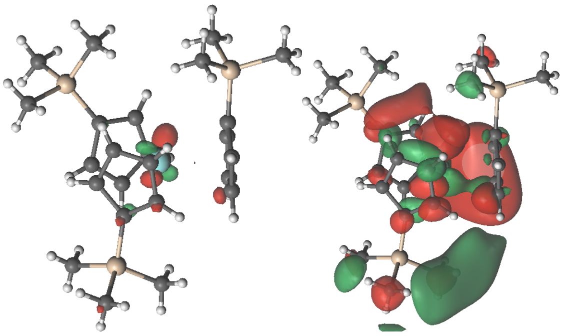

Supplementary Figure 53. Natural SOMO for state 8 in the CASSCF calculations for the crystalline geometry of **1** with a sphere of point charges. Isovalue = 0.04 (left) and 0.02 (right).

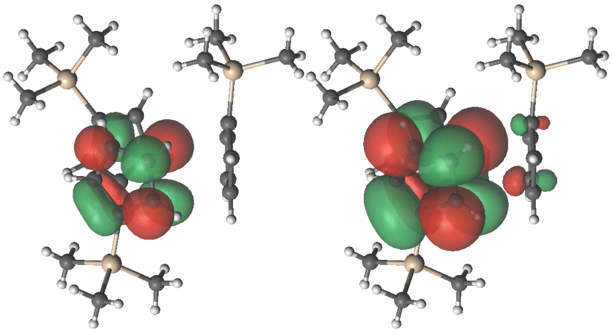

Supplementary Figure 54. Natural SOMO for state 9 in the CASSCF calculations for the crystalline geometry of **1** with a sphere of point charges. Isovalue = 0.04 (left) and 0.02 (right).

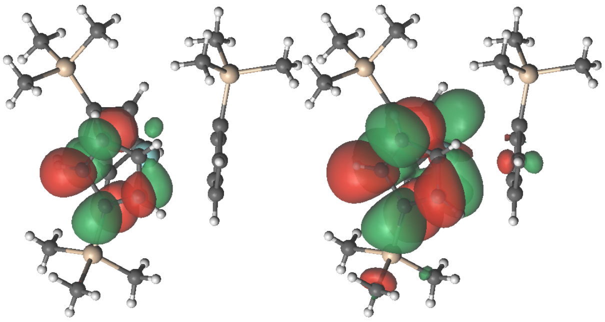

Supplementary Figure 55. Natural SOMO for state 10 in the CASSCF calculations for the crystalline geometry of **1** with a sphere of point charges. Isovalue = 0.04 (left) and 0.02 (right).

Supplementary Tables

Supplementary Table 1. Extracted phase memory time constants for **1** (THF), at X-band (**OP1-OP4**; Fig. 2a).

T (K)	**OP1** T_m (ns)	**x**	**OP2** T_m (ns)	**x**	**OP3** T_m (ns)	**x**	**OP4** T_m (ns)	**x**	$t(\pi/2)$ (ns)
5	2851.9	1.55	1971.5	1.55	2380.8	1.75	2606.3	1.86	256
10	2886.2	1.51	1924.9	1.47	2493.8	1.80	2481.2	1.91	256
15	2012.9	1.53	1998.0	1.50	1911.0	1.50	1894.5	1.50	256
20	2010.9	1.52	1961.5	1.54	1888.7	1.44	1875.2	1.50	256
30	1872.0	1.51	1861.0	1.53	1745.6	1.46	1725.8	1.45	256
40	1388.8	1.28	1497.6	1.36	1468.3	1.35	1445.0	1.35	256
50	1243.8	1.27	1142.8	1.20	1189.4	1.26	1190.8	1.27	256
60	1211.0	1.03	1116.3	1.17	939.9	1.10	937.5	1.15	64
80	507.2	1.00	467.1	1.00	510.9	1.00	497.7	1.00	64
100	844.7	1.00	515.2	1.00	810.0	1.00	755.6	1.00	64
120	693.2	1.00	580.3	1.00	560.9	1.00	484.2	1.00	64

Supplementary Table 2. Extracted phase memory time constants for single crystal **~2% 1@5** (**OP1-OP2**; Fig. 2b)

T (K)	OP1 (z) T_m (ns)	**x**	$t(\pi/2)$ (ns)	OP2 (x,y) T_m (ns)	**x**	$t(\pi/2)$ (ns)
5	2067.9	1.70	256	1480.5	1.50	128
7.5	1783.5	1.65	256	1082.6	1.40	128
10	1701.0	1.60	256	1121.5	1.43	128
15	1963.0	1.60	256	1359.2	1.42	128
20	1983.5	1.58	256	1330.9	1.30	128
30	1921.4	1.62	256	1355.3	1.28	128
40	1684.4	1.67	256	1483.4	1.27	128
50	1119.7	1.62	256	752.0	1.30	128
60	741.6	1.50	128	634.8	1.41	64
80	756.1	1.40	64	545.0	1.40	64
100	734.8	1.20	64	620.7	1.30	64
120	712.9	1.00	64	707.1	1.10	64
160	672.6	1.00	64	733.0	1.00	64
200	464.7	1.00	64	487.9	1.00	64
240	449.7	1.00	64	440.0	1.00	64
298	378.5	1.00	64	375.2	1.00	64

Supplementary Table 3. Extracted spin lattice relaxation time constants for **1** (THF) (**OP1-OP4**; Fig. 2a)

T (K)	**OP1**		**OP2**		**OP3**		**OP4**	
	T_1 (μs)	T_{SD} (μs)	T_1 (μs)	T_{SD} (μs)	T_1 (μs)	T_{SD} (μs)	T_1 (μs)	T_{SD} (μs)
5	41273.6	2598.7	26637.5	1994.6	22367	2201.2	24954	2314.6
10	38009.7	3100.4	21672.3	1770.9	27449.6		37952	3199.2
15	22780	1761.6	10083.6	293.4	14115.2		12002.8	1090.1
20	7056	41.1	7605.7		5988		7855.8	
30	2290		1155.6		1527.2		1852.8	
40	667.3		602.9		614.6		897	
50	327.2		249.2		272.9		338	
60	150.8		156.5		202.4		203	
80	61.5		55.6		115.7		71.2	
100	45.1		45.5		41.2		35.6	
120	11.2		13.2		18.3		18	

Supplementary Table 4. Extracted spin lattice relaxation time constants for **~2% 1@5** (**OP1-OP2**; Fig. 2b)

T (K)	**OP1** ($B_0 \parallel C_3$)		**OP2** ($B_0 \perp C_3$)	
	T_1 (μs)	T_{SD} (μs)	T_1 (μs)	T_{SD} (μs)
5	17951.8	4588.3	8465	540.5
7.5	15109.6	3750.3	9159.5	2677.6
10	14444.8	3750.3	9199.1	2965.7
15	11582.8	3200.7	7357.7	2085.2
20	7423.5	2159.3	4771.6	2346.8
30	1548		1716.6	1193.6
40	616.5		626.5	
50	287.2		320	
60	137.1		167.8	
80	63.2		59.7	
100	28.5		31.5	
120	16.7		13.1	
160	8.4		10.3	
200	2.8		4.7	
240	2.6		3.5	
298	2		1.5	

Supplementary Table 5. Rabi frequencies for **1** (THF) at B_0 = 349.8 mT (**OP1**; Fig. 2c)

Attenuation (dB)	Relative B_1 (a.u.)	Ω_R (MHz) at 40 K	Ω_R (MHz) at 120 K
1	5.62	26.69	-
3	4.47	21.19	21.46
5	3.55	17.01	17.23
7	2.82	11.90	11.97
10	2	9.05	9.15
13	1.41	6.53	6.61
16	1	4.74	4.83
20	0.63	3.23	3.22

Supplementary Table 6. Rabi frequencies for **1** (THF**)** at B_0 = 355.9 mT (**OP4**; Fig. 2c)

Attenuation (dB)	Relative B_1 (a.u.)	Ω_R (MHz) at 40 K	Ω_R (MHz) at 120 K
1	5.62	26.64	-
3	4.47	21.07	21.46
5	3.55	16.64	17.37
7	2.82	12.05	12.01
10	2	8.94	9.19
13	1.41	6.39	6.59
16	1	4.67	4.65
20	0.63	3.28	3.24
22	0.5	2.69	
24	0.4	2.21	

Supplementary Table 7. Rabi frequencies for **1** (THF) at B_0 = 352.2 mT (**OP3;** Fig. 2c)

Attenuation (dB)	Relative B_1 (a.u.)	Ω_R (MHz) at 40 K
1	5.62	26.60
3	4.47	21.17
5	3.55	17.10
7	2.82	11.90
10	2	8.98
13	1.41	6.47
16	1	4.74
20	0.63	3.36
24	0.4	2.17

Supplementary Table 8. Rabi frequencies for **~2% 1@5** (single crystal) at 347 mT (**OP1;** Fig. 2c; $B_0||C_3$).

Attenuation (dB)	Relative B_1 (a.u.)	Ω_R (MHz) at 120 K	Ω_R (MHz) at 298 K
1	5.62	30.74	29.04
3	4.47	24.65	23.8
5	3.55	19.52	18.54
7	2.82	15.86	14.64
8	2.51	12.69	14.64
11	1.79	9.51	9.02
13	1.41	7.56	7.32
15	1.12	5.85	5.61
17	0.89	4.63	4.64
20	0.63	3.66	3.41
22	0.5	2.92	2.68
24	0.4	2.19	

Supplementary Table 9. Rabi frequencies for **~2% 1@5** (single crystal) at B_0 = 349 and 352.7 mT (**OP2** and **OP4**; Fig. 2c; $B_0 \perp C_3$).

Att. (dB)	Rel. B_1 (a.u.)	Ω_R (MHz) at 120 K **OP2**	Ω_R (MHz) at 120 K **OP4**	Ω_R (MHz) at 298 K **OP2**	Ω_R (MHz) at 298 K **OP4**
3	4.47	10.23	10.25		
5	3.55	8.78		9.26	
7	2.82	7.12	7.11	8.51	8.51
8	2.51	6.18		7.35	
9	2.2	5.6	5.58	6.52	6.53
11	1.79	4.67		5.64	
13	1.41	3.9	3.91	4.56	4.59
15	1.12	3.23		3.89	
17	0.89	2.81	2.84	3.17	3.16

Supplementary Table 10. Basis sets used for geometry optimisations (20).

**Compound**	**Metal atom**	**Non-metal atoms**
**1**	old-DKH-TZVP	DKH-def2-TZVP
**2**	SARC-DKH-TZVP	DKH-def2-TZVP
**3**	SARC-ZORA-TZVP	ZORA-def2-TZVP
**4**	old-DKH-TZVP	DKH-def2-TZVP

Supplementary Table 11. Summary of DFT results for the crystal structure of **1**. Basis sets on all other atoms were DKH-def2-TZVP.

		A^{Y} (MHz)				Lödwin fractional spin density				*g*-values		
**Functional**	**Y basis set**	A_x	A_y	A_z	A_{iso}	**Y**	**Y(s)**	**Y(d)**	**Y(p)**	g_x	g_y	g_z
**PBE**	old-DKH-SVP	-90.4244	-90.9842	-95.0167	-92.1418	0.6165	0.0786	0.5203	0.0175	1.9909	1.9922	2.0026
	old-DKH-TZVP	-88.3904	-89.0201	-93.8617	-90.4241	0.6167	0.0751	0.5237	0.0180	1.9892	1.9907	2.0026
	old-DKH-TZVPP	-88.8710	-89.5021	-93.7129	-90.6953	0.6102	0.0757	0.5214	0.0139	1.9893	1.9908	2.0026
	ANO-RCC-DZP	-87.9309	-88.5608	-91.5312	-89.3409	0.5809	0.0716	0.4945	0.0146	1.9895	1.9910	2.0026
	Sapporo-DKH3-TZP-2012	-90.1572	-90.7353	-94.6198	-91.8374	0.6346	0.0758	0.5467	0.0117	1.9902	1.9916	2.0026
	Sapporo-DKH3-QZP-2012	-91.4170	-91.9986	-95.6088	-93.0081	0.6271	0.0762	0.5389	0.0113	1.9900	1.9914	2.0026
**BP86**	old-DKH-TZVP	-92.2487	-92.8690	-97.7665	-94.2948	0.6269	0.0719	0.5351	0.0199	1.9888	1.9903	2.0026
	Sapporo-DKH3-TZP-2012	-93.7947	-94.3650	-97.9639	-95.3745	0.6435	0.0722	0.5584	0.0130	1.9899	1.9913	2.0026
**B3LYP**	old-DKH-TZVP	-95.3484	-96.0436	-100.8021	-97.3980	0.6357	0.0730	0.5468	0.0159	1.9864	1.9881	2.0026
	Sapporo-DKH3-TZP-2012	-95.1732	-95.8114	-99.2390	-96.7412	0.6519	0.0741	0.5680	0.0105	1.9879	1.9894	2.0027
**TPSSh**	old-DKH-TZVP	-88.9258	-89.5573	-92.6207	-90.3679	0.6404	0.0748	0.5446	0.0210	1.9890	1.9904	2.0026
	Sapporo-DKH3-TZP-2012	-85.8018	-86.3833	-87.4726	-86.5525	0.6560	0.0746	0.5687	0.0140	1.9900	1.9912	2.0026
**M06**	old-DKH-TZVP	-95.2193	-95.9954	-103.7934	-98.3360	0.6527	0.0905	0.5344	0.0279	1.9869	1.9890	2.0027
	Sapporo-DKH3-TZP-2012	-83.1192	-83.9206	-90.9025	-85.9808	0.6868	0.0906	0.5755	0.0280	1.9879	1.9900	2.0026
**PBE0**	old-DKH-TZVP	-92.2376	-92.8894	-94.7304	-93.2858	0.6479	0.0748	0.5524	0.0207	1.9881	1.9895	2.0023
	Sapporo-DKH3-TZP-2012	-92.6475	-93.2515	-94.1271	-93.3420	0.6639	0.0746	0.5763	0.0140	1.9892	1.9905	2.0024
**Average**	-	-91(3)		-95(4)	-92(4)	0.64(2)	0.077(6)	0.54(2)	0.017(5)	1.990(1)		2.0026(1)

Supplementary Table 12. DFT results using ZORA relativistic Hamiltonian for the crystal structure of **1**. Basis sets on all other atoms were ZORA-def2-TZVP.

Functional	Y basis set	A_x	A_y	A_z	A_{iso}	g_x	g_y	g_z
**PBE**	old-ZORA-SVP	-102.4753	-103.5730	-105.1821	-103.7435	1.9912	1.9925	2.0026
	old-ZORA-TZVPP	-97.3283	-98.3425	-100.1217	-98.5975	1.9911	1.9925	2.0026

Supplementary Table 13. DFT results for the crystal structure of **1** with point charges at the Y and K lattice sites.

Functional	Y basis set	A_x	A_y	A_z	A_{iso}	g_x	g_y	g_z
**PBE**	old-DKH-TZVP	-98.4899	-99.3885	-103.2942	-100.3909	1.9874	1.9893	2.0023

Supplementary Table 14. Hyperfine coupling parameters from DFT for **1-4**, calculated with PBE, DKH and the appropriate def2-TZVP basis set.

Compound/Structure	Metal basis set	A^Y (MHz)				Lödwin fractional spin density				*g*-values		
		A_x	A_y	A_z	A_{iso}	Metal	Metal(s)	Metal(d)	Metal(p)	g_x	g_y	g_z
**1** (crystalline)	old-DKH-TZVPP	-88.3904	-89.0201	-93.8617	-90.4241	0.6167	0.0751	0.5237	0.0180	1.9892	1.9907	2.0026
**1** (optimised)	old-DKH-TZVPP	-70.1384	-71.2243	-75.6440	-72.3355	0.5716	0.0630	0.4929	0.0157	1.9900	1.9946	2.0025
**2** (crystalline)	SARC-DKH-TZVP	989.4181	991.8951	1004.8720	995.3951	0.6237	0.1040	0.5062	0.0165	1.9664	1.9716	2.0021
**2** (optimised)	SARC-DKH-TZVP	917.3997	920.1777	933.4989	923.6921	0.5977	0.0921	0.4921	0.0159	1.9684	1.9734	2.0023
**3** (crystalline)	SARC-DKH-TZVP	311.2075	312.8486	324.9602	316.3388	0.6780	0.0871	0.5792	0.0072	1.9664	1.9683	2.0021
**3** (optimised)	SARC-DKH-TZVP	336.9145	338.6541	350.3511	341.9732	0.6801	0.0881	0.5805	0.0071	1.9644	1.9662	2.0019
**4** (crystalline)	old-DKH-TZVP	552.1476	556.8163	589.1498	566.0379	0.7978	0.1292	0.6581	0.0104	1.9833	1.9845	2.0017
**4** (optimised)	old-DKH-TZVP	537.0734	541.3868	572.7468	550.4023	0.7684	0.1237	0.6338	0.0109	1.9845	1.9856	2.0017

Supplementary Table 15. Occupation numbers in the state averaged CASSCF calculation for the crystalline geometry of **1**.

**Orbital**	130	131	132	133	134	135	136	137
**Occupation**	1.98	1.98	1.98	0.10	0.11	0.01	0.10	0.10
**Orbital**	138	139	140	141	142	143	144	145
**Occupation**	0.11	0.01	0.10	0.10	0.10	0.10	0.01	0.10

Supplementary Table 16. Relative energies of the 10 lowest states for CASSCF calculations of **1**.

**State**	**Energy (cm^{-1})**	
	**Crystalline**	**Crystalline (point charges)**
1	0	0
2	9242	4698
3	9860	10301
4	12246	14930
5	13559	18401
6	16158	19634
7	16909	20896
8	18814	26665
9	33572	28531
10	34142	31136

Supplementary Table 17. Contributions > 3% to the natural SOMO for state 1 in **1** (total for atomic angular momenta).

**AO**	**Contribution (%)**
Y(4d)	50.1
Y(5s)	5.3
**Total**	
Y	57.7
Ligand	42.3

Supplementary Table 18. Contributions > 3% to the natural SOMO for state 2 in **1** (total for atomic angular momenta).

AO	Contribution (%)
H38(2s)	5.2
H32(2s)	4.0
H36(2s)	3.7
Y(4d)	3.4
H39(2s)	3.3
Y(6s)	3.2
H35(2s)	3.2
**Total**	
Y	10.6
Ligand	89.4

Supplementary Table 19. Contributions > 3% to the natural SOMO for state 3 in **1** (total for atomic angular momenta).

AO	Contribution (%)
H38(2s)	6.3
H19(2s)	5.1
H21(2s)	4.2
H8(2s)	4.0
H39(2s)	3.9
H36(2s)	3.7
H9(2s)	3.3
H16(2s)	3.3
H18(2s)	3.2
H37(2s)	3.1
**Total**	
Y	2.8
Ligand	97.2

Supplementary Table 20. Contributions > 3% to the natural SOMO for state 4 in **1** (total for atomic angular momenta).

**AO**	**Contribution (%)**
H6(2s)	6.7
H5(2s)	5.9
H1(2s)	5.3
H16(2s)	5.2
H2(2s)	4.7
H15(2s)	4.7
H14(2s)	4.5
**Total**	
Y	3.9
Ligand	96.1

Supplementary Table 21. Contributions > 3% to the natural SOMO for state 5 in **1** (total for atomic angular momenta).

**AO**	**Contribution (%)**
Y(6p)	7.7
H25(2s)	7.7
H11(2s)	5.2
Y(7p)	4.7
H22(2s)	4.1
H28(2s)	3.8
H10(2s)	3.5
H26(2s)	3.1
H35(2s)	3.0
**Total**	
Y	15.8
Ligand	84.2

Supplementary Table 22. Contributions > 3% to the natural SOMO for state 6 in **1** (total for atomic angular momenta).

**AO**	**Contribution (%)**
H31(2s)	7.9
H32(2s)	7.8
H14(2s)	4.2
H12(2s)	3.7
H16(2s)	3.6
H18(2s)	3.4
**Total**	
Y	6.9
Ligand	93.1

Supplementary Table 23. Contributions > 3% to the natural SOMO for state 7 in **1** (total for atomic angular momenta).

**AO**	**Contribution (%)**
H35(2s)	8.4
H36(2s)	4.3
H24(2s)	3.9
H22(2s)	3.4
H9(2s)	3.2
**Total**	
Y	5.9
Ligand	94.1

Supplementary Table 24. Contributions > 3% to the natural SOMO for state 8 in **1** (total for atomic angular momenta).

AO	Contribution (%)
H2(2s)	5.5
H21(2s)	4.7
H13(2s)	4.4
H15(2s)	4.1
H27(2s)	3.7
H5(2s)	3.4
H7(2s)	3.3
Y(6d)	3.3
H8(2s)	3.3
H14(2s)	3.1
H20(2s)	3.1
**Total**	
Y	7.4
Ligand	92.6

Supplementary Table 25. Contributions > 3% to the natural SOMO for state 9 in **1** (total for atomic angular momenta).

AO	Contribution (%)
Y(4d)	59.5
Y(5d)	20.3
**Total**	
Y	82.6
Ligand	17.4

Supplementary Table 26. Contributions > 3% to the natural SOMO for state 10 in **1** (total for atomic angular momenta).

AO	Contribution (%)
Y(4d)	58.2
Y(5d)	21.2
**Total**	
Y	82.7
Ligand	17.3

Supplementary Table 27. Occupation numbers in the state averaged CASSCF calculation for the crystalline geometry of **1** with a sphere of point charges.

**Orbital**	131	132	133	134	135	136
**Occupation**	1.95	1.95	0.15	0.10	0.11	0.10

**Orbital**	137	138	139	140	141	142
**Occupation**	0.14	0.10	0.10	0.10	0.10	0.10

Supplementary Table 28. Contributions > 3% to the natural SOMO for state 11 in **1** with a sphere of point charges (total for atomic angular momenta).

AO	Contribution (%)
Y(4d)	48.5
Y(5s)	5.6
**Total**	
Y	56.5
Ligand	43.5

Supplementary Table 29. Contributions > 3% to the natural SOMO for state 2 in **1** with a sphere of point charges (total for atomic angular momenta).

**AO**	**Contribution (%)**
H18(2s)	25.9
H10(2s)	10.2
H26(2s)	6.3
H17(2s)	5.7
H19(2s)	5.2
H18(1s)	4.5
C7(2s)	4.0
Y(4d)	3.9
H25(2s)	3.0
**Total**	
Y	10.7
Ligand	89.3

Supplementary Table 30. Contributions > 3% to the natural SOMO for state 3 in **1** with a sphere of point charges (total for atomic angular momenta).

**AO**	**Contribution (%)**
H29(2s)	14.3
H28(2s)	12.5
Y(6p)	5.6
Y(5p)	4.0
Y(4d)	3.8
Y(7p)	3.6
H27(2s)	3.2
H34(2s)	3.0
**Total**	
Y	19.9
Ligand	80.1

Supplementary Table 31. Contributions > 3% to the natural SOMO for state 4 in **1** with a sphere of point charges (total for atomic angular momenta).

**AO**	**Contribution (%)**
H11(2s)	12.6
H25(2s)	8.1
H24(2s)	7.4
H37(2s)	6.7
Y(6p)	5.2
H38(2s)	4.3
H39(2s)	3.3
Y(6s)	3.2
**Total**	
Y	19.5
Ligand	80.5

Supplementary Table 32. Contributions > 3% to the natural SOMO for state 5 in **1** with a sphere of point charges (total for atomic angular momenta).

**AO**	**Contribution (%)**
Y(4d)	8.5
H3(2s)	8.2
H2(2s)	6.8
H1(2s)	5.1
**Total**	
Y	14.9
Ligand	85.1

Supplementary Table 33. Contributions > 3% to the natural SOMO for state 6 in **1** with a sphere of point charges (total for atomic angular momenta).

**AO**	**Contribution (%)**
Y(4d)	32.9
H3(2s)	3.2
**Total**	
Y	40.2
Ligand	59.8

Supplementary Table 34. Contributions > 3% to the natural SOMO for state 7 in **1** with a sphere of point charges (total for atomic angular momenta).

**AO**	**Contribution (%)**
H16(2s)	8.2
H15(2s)	7.7
H21(2s)	6.0
H14(2s)	5.0
H22(2s)	3.3
H8(2s)	3.2
Y(4d)	3.2
**Total**	
Y	7.5
Ligand	92.5

Supplementary Table 35. Contributions > 3% to the natural SOMO for state 8 in **1** with a sphere of point charges (total for atomic angular momenta).

**AO**	**Contribution (%)**
H25(2s)	6.4
H35(2s)	5.2
H36(2s)	4.3
Y(4d)	4.1
H34(2s)	3.8
H39(2s)	3.5
H38(2s)	3.4
Y(6d)	3.2
**Total**	
Y	12.0
Ligand	88.0

Supplementary Table 36. Contributions > 3% to the natural SOMO for state 9 in **1** with a sphere of point charges (total for atomic angular momenta).

**AO**	**Contribution (%)**
C17(2p)	23.2
C18(2p)	21.5
C19(2p)	9.6
Y(4d)	5.2
C21(3d)	4.4
C18(3d)	4.3
C17(3d)	4.0
C19(3d)	3.7
Y(5f)	3.7
C20(3d)	3.2
**Total**	
Y	11.9
Ligand	88.1

Supplementary Table 37. Contributions > 3% to the natural SOMO for state 10 in **1** with a sphere of point charges (total for atomic angular momenta).

**AO**	**Contribution (%)**
C20(2p)	26.6
C21(2p)	22.2
C19(2p)	7.3
C17(2p)	4.8
C20(3d)	3.7
C19(3d)	3.6
C21(3d)	3.6
C17(3d)	3.3
**Total**	
Y	7.9
Ligand	92.1

Supplementary Methods

All manipulations and syntheses were conducted with rigorous exclusion of air and water using standard Schlenk line and glovebox techniques under an argon or dinitrogen atmosphere. Solvents were sparged with UHP argon and dried by passage through columns containing Q-5 and molecular sieves prior to use. 2.2.2-Cryptand (4,7,13,16,21,24-hexaoxa-1,10-diazabicyclo[8.8.8]hexacosane, Aldrich) was placed under vacuum (10^{-3} Torr) for 12 h before use. Anhydrous $LnCl_3$ (Ln = Y, Yb),[1] $KC_5H_4SiMe_3$,[2] $(C_5H_4SiMe_3)_3Ln$ (Ln = Y,[3] Yb[4]), potassium graphite,[5] and [K(2.2.2-cryptand)][$(C_5H_4SiMe_3)_3Ln$] (Ln = Y (**1**),[6] Yb[4]) were prepared according to literature procedures. Magnetically dilute complex **~2% 1@5** was obtained by recrystallization from a solution mixture that contained the appropriate amounts of isostructural [K(2.2.2-cryptand)][$(C_5H_4SiMe_3)_3Y$] (**1**) and [K(2.2.2-cryptand)][$(C_5H_4SiMe_3)_3Yb$] compounds, the latter acting as the diamagnetic matrix. The molecular structure of compound **1** is presented in Supplementary Figure 1.

Synthesis of single crystals of ~2% 1@5 (2% doping level): In an argon atmosphere glovebox, [K(2.2.2-cryptand)][$(C_5H_4SiMe_3)_3Y$] (2 mg, 0.002 mmol) and [K(2.2.2-cryptand)][$(C_5H_4SiMe_3)_3Yb$] (100 mg, 0.09 mmol) were dissolved in 1 mL of THF to afford a dark green solution. Dark green single crystals of **~2% 1@5** were grown over two days at −35 °C from slow vapor diffusion of Et_2O into the THF solution.

Continuous-wave EPR Measurements. Continuous-wave (CW) electron paramagnetic resonance (EPR) spectra of solution samples of **1** (Figs. 2a) were recorded on either a Bruker EMX 300 or a Bruker ElexSys E580 EPR spectrometer operating at X-band (*ca.* 9.4-9.8 GHz) and variable temperatures. CW EPR spectra of oriented single crystals of **~2% 1@5** (Supplementary Figure 3) were collected with a Bruker ElexSys E580 instrument operating at *ca.* 9.7 GHz and varied temperatures, and equipped with a goniometer that allowed controlled crystal rotation. An identical setup was used to measure the same crystal by echo-detected pulsed-EPR methods (see below). Crystals were indexed by X-ray crystallography to determine the precise orientation of the crystallographic axes. Similarly to **1** and its Yb analogue, compound **~2% 1@5** crystallises in a Monoclinic ($P2_{1/C}$) space group. Its single crystal unit cell parameters: a = 16.22 Å, b = 24.79 Å, c = 14.04 Å, α = 90°, β = 90.57°, γ = 90°, and V = 5344, are similar to those of **1** (a = 15.8992(9) Å, b = 24.2949(14) Å, c = 13.7571(8) Å, α = 90°, β = 91.0257(8)°, γ = 90°, V = 5313.1(5) Å^3),[6] and diamagnetic host [K(2.2.2-cryptand)][$(C_5H_4SiMe_3)_3Yb$] (a = 15.9471(5) Å, b = 24.2284(8) Å, c = 13.8243(5) Å, α = 90°, β = 91.1563(4)°, γ = 90°, and V = 5340.2(3) Å^3).[4] We note that molecules pack in the crystal in different ways, being classified in two groups: **A** and **B**, whose C_3 axes (that is the axis perpendicular to the plane made by the centroids of the three Cp' ligands bound to Y^{2+}) are orthogonal. As such, we have chosen to rotate the crystal around the C_3 axis of one of such molecules, in order to access such an orientation that

molecules **B** would have their C_3 axis parallel to the static B_0 field (Supplementary Figure 3), while molecules **A** remained aligned with C_3 perpendicular to B_0. This allowed determination of both g_z and $g_{x,y}$ components at the same time (Table 2, main text). Spectra were simulated using the EasySpin software.[7]

Pulsed EPR Measurements. Pulsed EPR spectra were recorded with a Bruker ElexSysE580 instrument equipped with either a MD5 or a MD4 resonator, and operating at *ca*. 9.7 GHz and various temperatures. Solution samples of different concentrations (2, 5 and 10 mM in THF) were investigated to check reproducibility and to achieve an acceptable signal-to-noise response in HYSCORE and ENDOR experiments. Single crystal measurements I involved an identical instrumental set-up as described above.

Echo-detected EPR. The echo-detected field–swept (EDFS) spectra (Figs. 2a, 2b and Supplementary Figure 4) were recorded with a two-pulse primary Hahn-echo sequence ($\pi/2$ - τ - π - τ - *echo*),[8] with microwave pulse lengths of 16 and 32 ns, respectively, a fixed delay time τ = 300 ns, and with the variation of the static B_0 magnetic field.

Phase Memory Time (T_m). Electron spin echo envelope modulation (ESEEM) measurements involved monitoring the echo intensity generated with a primary Hahn-echo sequence as a function of τ. A similar pulse sequence was used to measure the phase memory time, T_m, with the difference that longer pulse durations (up to 512 nm) were necessary to suppress possible ^{1}H nuclear modulation effects in the echo decays (Supplementary Figure 5). T_m was determined by least squares fitting of the experimental echo decay data using a stretched exponential function with a solver based on the Levenberg-Marquardt algorithm.

The fitting function used was:

$$Y(2\tau) = Y(0)e^{(-2\tau/T_m)X} \quad \text{(Equation 1)}$$

or, for strongly modulated data,

$$Y(2\tau) = Y(0)e^{(-2\tau/T_m)X}(1 + k\sin(\omega t + \Phi)) \quad \text{(Equation 2)}$$

where k is the modulation depth, ω is the Larmor angular frequency of a nucleus I coupled to the electron spin, ϕ is the phase correction, X is the stretching parameter, $Y(2\tau)$ is the echo integral for a pulse separation τ, and $Y(0)$ is the echo intensity extrapolated to $\tau = 0$.[9-12]

The extracted T_m times for **1** and **~2% 1@5** are given in Supplementary Tables 1 and 2. Owing to the 2p-ESE decays being dependent on experimental conditions, with longer (more selective) pulses resulting in longer relaxation decays (Supplementary Figure 6), comparison between the extracted T_m values at those temperatures must be regarded with caution.

Spin–lattice Relaxation Time (T_1). Spin-lattice relaxation time data (Supplementary Figures 7 and 8) were acquired with a standard magnetisation inversion recovery sequence, π–t–$\pi/2$–τ–π–τ–*echo*,[8] with t_π = 32 ns, τ = 320 ns, and variable t. The spin-lattice relaxation time constant, T_1, was determined by fitting the experimental data to the following biexponential decay function:

$$Y(t) = Y(0) + Y_1 e^{(-t/T_1)} + Y_{SD} e^{(-t/T_{SD})} \quad \text{(Equation 3)}$$

where Y_1 and Y_{SD} are the amplitudes, and T_{SD} is the spectral diffusion time constant,[11] giving the results presented in Figs. 2e, 2f and S9, and Supplementary Tables 3 and 4. The presence of two decays is commonly attributed to the occurrence of both spectral diffusion and spin-lattice relaxation of which the latter is usually assigned as being the slower process.[12] We notice that the magnetization recovery curves do not reach full saturation below 15 K, indicating that the T_1 spin-lattice relaxation time is very long. Fitting such curves to an exponential model is likely to introduce some inaccuracy in the determination of the T_1 values at these temperatures.

Transient Nutation Experiments (Rabi oscillations). The transient nutation data (Figs. 2c and 2d, and Supplementary Figures 11 to 22) were acquired with a three-pulse nutation

sequence, t_p–t_w–π/2–τ–π–τ–*echo*.[8,13] The length of the tipping pulse, t_p, pulse was varied in 2 ns increments, whilst those of the pulses π/2 and π were kept fixed at the optimal values needed to generate a maximum echo intensity, for t_p = 0. The t_w and τ delays were kept constant at 6 μs (chosen to be much longer than T_m) and 200 ns, respectively. The Rabi frequency, Ω_R, was determined by zero-filling the Rabi oscillation curves, followed by Fast Fourier Transform (FFT). Ω_R is expected to vary linearly with B_1, as opposed to nuclear modulations that are insensitive to the strength of B_1 (Supplementary Figures 16 and 23) (11).

HYSCORE (Hyperfine sub–level correlation) Measurements. The HYSCORE spectra were recorded with a four–pulse sequence, π/2–τ–π/2–t_1–π–t_2–π/2–*echo*,[8] with pulses $\pi/2$ and π of 16 and 32 ns, respectively, and fixed τ (130, 200 or 400 ns). Times t_1 and t_2 were varied from 100 to 5200 ns in increments of 20 ns. 256 data points were collected in both dimensions. A four-step phase-cycle procedure was used to eliminate unwanted echo contributions. Fourier transformation of the data in both directions yielded 2D (υ_1,υ_2) spectra (Figure 3a,c and Supplementary Figures 26 and 27) in which the nuclear cross-peaks (i.e. peaks that correlate nuclear frequencies from opposite spin-manifolds) of the ^{1}H and ^{13}C nuclei appeared in the (+,+) quadrant of the (υ_1,υ_2) map, at separations equivalent with the corresponding hyperfine coupling frequencies (weak coupling regime: $2|\upsilon_n|>|A|$).[8] The contour lineshape of the cross peaks, and their displacement from the anti-diagonal about the nuclear Larmor frequency (υ_n), relate to the magnitude and anisotropy of the hyperfine couplings, and thus analysis of the HYSCORE spectra allows to determine such parameters. Spectra modelling with EasySpin[7] has assumed that the total hyperfine coupling matrix (**A**) for a given ^{13}C nucleus *n* is determined by the spin density at nucleus *n* ($\mathbf{A}^{Cn}$), and the point dipole (through space) interactions with spin density at other atoms k ($\mathbf{A}^{dip}$), according to the equation: $\mathbf{A} = \mathbf{A}^{Cn} + \mathbf{A}^{dip}$.[10] $\mathbf{A}^{Cn}$ relates directly to the covalency. A^{dip} is given by Equation (4):

$$A^{\text{dip}} = \frac{\mu_0}{4\pi h}\beta_e\beta_n \sum_k \rho_k \frac{3(\mathbf{g}.\mathbf{n_k})(\tilde{\mathbf{n}}_\mathbf{k}.g_n\mathbf{1}) - \mathbf{g}.g_n\mathbf{1}}{r_k^3} \qquad \text{(Equation 4)}$$

where **g** and g_n**1** are the electron and nuclear **g** (3x3) matrices (g_n is a scalar; **1** is the unit matrix), β_e and β_n are the electron and nuclear magnetons, ρ_k is the electron spin population at atom k ($0 \leq \rho_k \leq 1$), r_k is $n \ldots k$ distance, $\mathbf{n}_k$ and $\tilde{\mathbf{n}}_k$ are the $n \ldots k$ unit vector expressed in the molecular frame (a column vector) and its transpose, h is the Plank's constant, and μ_0 is the vacuum permittivity. It is also assumed that g_z lies along the C_3 unique axis (Supplementary Figure 24), and the dominant spin density is located at the yttrium ion (ρ_Y = 1). $\boldsymbol{A}^{\text{dip}}$ is then calculated for each unique carbon position in the Cp' ligands, using the crystallographic coordinates of the atoms. Simulations considering only $\mathbf{A}^{\text{dip}}$ do not produce satisfactory results (Supplementary Figure 25). By contrary, addition of $\mathbf{A}^{\text{Cn}}$ to $\mathbf{A}^{\text{dip}}$ allows to reproduce the experimental data (Figs. 3a,b and Supplementary Figure 26). As the non-metal frontier orbitals of $[Y(Cp')_3]^-$ comprise the π-systems of the Cp' rings, the bulk of any spin density transferred from the metal ion will be in these C 2pπ - orbitals.[10,15] Thus, for each matrix $\boldsymbol{A}^{\text{Cn}}$ (assumed to be axial) we fix the unique axis to be oriented along the 2pπ direction (*i.e.* in the molecular xy plane), which allows to determine A_z and $A_{x,y}$ per each C site (Table 2, main text). The $2p_\pi$ spin population (ρ_p) at the individual carbon positions can be derived from Equation (5):[10]

$A_{\parallel}$- $A_{\perp}$ **=** $6/5\rho_p P_p$ (Equation 5)

where P_p is the electron nuclear dipolar coupling parameter for unit population (ρ_p = 1) of a ^{13}C 2p orbital. Using the theoretical value of P_p = 268 MHz[15], we derive C $2p_\pi$ spin populations of ρ_p = 0.008 and 0.002, i.e. 0.8% at C2 and C5, and 0.2%, at C3 and C4.

Modelling of ^{1}H HYSCORE region for **1** involved a similar approach. We initially calculated the point dipolar ^{1}H hyperfine constants for all protons of the cyclopentadienyl rings, and all protons of the methyl groups supposed to be close to the Y(II) ion. This calculation failed to

reproduce the experimental data. We then added a contribution from the C $2p_\pi$ spin density on the Cp' ligands, which can occur via spin polarisation of the C-H bond.[10,16] Generally, the hyperfine coupling of an α-proton in a π radical has its principal values oriented with the smallest component along the C-H vector, one along the $2p_\pi$ direction, and the largest component orthogonal to the $2p_\pi$ and C-H directions.[17] As the C-H bonds of the Cp' rings are in the molecular xy plane, we expect the largest component to be oriented along the molecular z-axis (C_3 axis). The hyperfine matrix takes the form [$a_H/2$, a_H, $3a_H/2$], where a_H is the isotropic component, expected to have a negative sign. Best simulation of the ^1H HYSCORE data was achieved with a_{iso} = -0.7 MHz (Figs. 3c,d and Supplementary Figure 27). The isotropic hyperfine constant a_H at the α-proton relates to the spin density in the asociated C $2p_\pi$ orbital by the simple McConnell relationship: $a_{iso} = Q_{CH}*\rho_{p,}$, where Q_{CH} is the ^1H hyperfine coupling expected to be observed for $\rho_p = 1$. With Q_{CH} = - 84 MHz (determined from studies of Cp radicals)[17] and a_{iso} = -0.7 MHz, we get ρ_p = 0.00833 (**0.83 %**) for $C^{2,5}$ in excellent agreement with ρ_p = 0.008 from analysis of the ^{13}C data. This gives a total of ~ 6 % spin population on the three Cp' rings.

ENDOR (Electron nuclear double resonance) Measurements. Davies-ENDOR data (Supplementary Figure 28) were acquired with the standard pulse sequence, π–πRF–$\pi/2$–τ–π–τ–*inverted echo,*[19] with microwave pulses π/2 and π of 128 and 256 ns, respectively. Mims-ENDOR data (Supplementary Figure 29) were recorded by using a stimulated-echo sequence, $\pi/2$–τ–$\pi/2$–πRF–π/2–τ–*stimulated echo,*[19] based on three non-selective $\pi/2$ pulses (16 ns). In both cases, a radiofrequency pulse πRF of 12 μs was used. In order to correct the effect of potential blind spots, Mims-ENDOR spectra were collected at different inter-pulse delays, τ (200-600 ns). Spectra were simulated using Stoll's Easy Spin software,[26] yielding a_{iso} = -0.7 MHz for $H^{2/5}$ and a_{iso} = -0.27 MHz for $H^{3/4}$, which are in good agreement with the HYSCORE data.

DFT calculations. All DFT calculations were performed using ORCA 4.0.0.2 (18) with the unrestricted Kohn-Sham formalism on the *S* = 1/2 ground state of the anions in **1-4**. For geometry optimisations we started with the crystal structures, employed the PBE functional with the second order DKH transformation for the relativistic Hamiltonian, used the RI approximation for both the Coulomb and exchange integrals (using the SARC/J auxiliary basis) and Grimme's D3BJ dispersion corrections (19), along with tight SCF convergence criteria (TIGHTSCF, Grid3, FinalGrid5); basis sets are given in Supplementary Table 10. Only for **3** ($[La(Cp')_3]^-$) did we have problems with such an optimisation strategy, which could be resolved using the ZORA relativistic Hamiltonian.

To calculate the hyperfine coupling, most calculations employed the second order DKH transformation for the relativistic Hamiltonian, however we also checked that our results were consistent when employing the ZORA Hamiltonian; in both cases picture change effects were accounted for by setting the "picturechange" flag to "true". In all cases the non-metal atoms were described with the appropriate def2-TZVP basis set (either DKH-def2-TZVP or ZORA-def2-TZVP) (20). All calculations employed the RI approximation for both the Coulomb and exchange integrals where appropriate, employing the SARC/J auxiliary basis sets. To explore the metal atom basis set and exchange-functional dependence of these calculations, we performed hyperfine calculations for the crystal structure of **1** (Supplementary Table 11), finding an isotropic Y hyperfine coupling of *ca.* -100 MHz in all cases. We also found no significant changes using the ZORA Hamiltonian (Supplementary Table 12), nor when accounting for the electrostatic potential of the crystalline environment by using a sphere of 30 Å radius of point charges located at the K and Y lattice sites, with charges of +1 and -1, respectively (Supplementary Table 13).

As there was no significant effect on the obtained hyperfine parameters for the anion in **1** upon change of metal basis set, functional, relativistic Hamiltonian or inclusion of the crystalline electrostatic potential, we subsequently calculated the hyperfine parameters for

both crystalline and optimised structures for the anions of **1-4** in the gas phase using the PBE functional, the appropriate def2-TZVP basis set, and the DKH Hamiltonian (Supplementary Table 14). Overall we find excellent agreement with the experimental data (Table 1 in main text), where all metal hyperfine coupling parameters and the *g*-values are nearly isotropic.

The orbital breakdown of the spin densities for **2-4** are remarkably similar to that described in the main text for **1**; only 50 – 80% of the spin density is located on the metal atom, in predominantly s and d functions (Supplementary Table 14). Despite the significant d-component and "d_{z^2}" appearance of the spin density (Figure S30), the anisotropies of the calculated metal hyperfines ($|A_z^Y - A_{x,y}^Y|/|A_{iso}^Y|$) are only on the order of 1 – 7% (Supplementary Table 14).

CASSCF calculations. State-averaged CASSCF calculations for the anion in **1** were performed with MOLCAS 8.0 (21). We used basis sets from the ANO-RCC library (22,23) with VTZP quality for the Y ion, VDZP quality for the Cp ring carbon atoms, and VDZ quality for all other atoms. The two electron integrals were Cholesky decomposed with a threshold of 10^{-8}. We employed an active space of 7 electrons in 16 orbitals (Supplementary Figures 31 to 33 and Supplementary Table 15), which was optimised for the 10 lowest-lying states (Supplementary Table 16). The natural orbitals (SOMOs) for each root were obtained by diagonalising the state-specific first-order density matrix (Supplementary Figures 34 to 43 and Supplementary Tables 17 to 26). Our calculations with a sphere with radius 30 Å of point charges at the K and Y lattice sites with +1 and -1 charges, respectively, yielded the appropriate active space as 5 electrons in 12 orbitals (Supplementary Figures 44 and 45 and Supplementary Table 27). The corresponding natural orbitals (SOMOs) for the ground state are practically identical to the gas-phase result (Supplementary Figure 46 and Supplementary Table 28), and states 2 – 5 are also diffuse ligand-based functions

(Supplementary Figures 47 to 50 and Supplementary Tables 29 to 32). However, state 6 at 19,634 cm^{-1} has significant Y 4d character (Supplementary Figure 51 and Supplementary Table 33), which is quite different to the gas-phase calculation, and thus the electrostatic crystalline potential has lowered the first 4d function by *ca.* 10,000 cm^{-1}. States 7 and 8 are diffuse ligand functions like for the gas-phase results (Supplementary Figures 52 and 53 and Supplementary Tables 34 and 35), however now states 9 and 10 are π* orbitals on the Cp' ring that is proximate to the K^+ counter ion (Supplementary Figures 54 and 55 and Supplementary Tables 36 and 37); these charge transfer states are now much lower in energy due to the stabilisation from the positive charge of the K^+ cation. Despite these differences in the higher energy states, the ground state SOMO and the diffuse character of the low-lying ligand-based excited states remains, and appears to be an intrinsic feature of this molecule.

Supplementary References

1. Taylor, M. D. *Chem. Rev.* **1962**, *62*, 503-511.
2. Peterson, J. K.; MacDonald, M. R.; Ziller, J. W.; Evans, W.J. *Organometallics* **2013**, *32*, 2625-2631.
3. MacDonald, M. R.; Ziller, J. W.; Evans, W. J. *J. Am. Chem. Soc.* **2011**, *133*, 15914-15917.
4. Fieser, M. E.; MacDonald, M. R.; Krull, B. T.; Bates, J. E.; Ziller, J. W.; Furche, F.; Evans, W. J. *J. Am. Chem. Soc.* **2015**, *137*, 369-382.
5. Bergbreiter, D. E; Killough, J. M. *J. Am. Chem. Soc.* **1978**, *100*, 2126-2134.
6. MacDonald, M. R.; Bates, J. E.; Ziller, J. W.; Furche, F.; Evans, W. J. *J. Am. Chem. Soc.* **2013**, *135*, 9857-9868.
7. S. Stoll, R. D. Britt, *Phys. Chem. Chem. Phys.*, **2009**, *11*, 6614-6625.
8. A. Schweiger, J. Jeschke, Principles of Pulse Electron Paramagnetic Resonance, Oxford University Press (2001).

9. C. J. Wedge, G. A. Timco, E. T. Spielberg, R. E. George, F. Tuna, S. Rigby, E. J. L. McInnes, R. E. P. Winpenny, S. J. Blundell, and A. Ardavan, *Phys. Rev. Lett.* **2012**, *108*, 107204/1-107204/5.

10. A. Formanuik, A.-M. Ariciu, F. Ortu, R. Beekmeyer, A. Kerridge, F. Tuna, E. J. L. McInnes, D. Mills, *Nature Chem.* **2017**, *9*, 578-583.

11. K. S. Pedersen, A.-M. Ariciu, A. McAdams, H. Weihe, J. Bendix, F. Tuna, S. Piligkos, *J. Am. Chem. Soc.* **2016**, *138*, 5801-5804.

12. K. Bader, M. Winkler, J. van Slageren, *Chem. Commun.* **2016**, *52*, 3623-3626.

13. J. Yang, Y. Wang, Z. Wang, X. Rong, C. K. Duan, J. H. Su, J. Du, *J. Phys. Rev. Lett.*, **2012**, *108*, 230501.

14. M. J. Graham, J. M. Zadrozny, M. Shiddiq, J. S. Anderson, M. S. Fataftah, S. Hill, D. E. Freedman, *J. Am. Chem. Soc.* **2014**, *136*, 7623.

15. J. R. Morton, K. F. Preston, *J. Magn. Reson.*, **1978**, *30*, 577-582.

16. N. M. Atherton. Principles of electron spin resonance, Ellis Horwood Ltd (1993).

17. F. Gerson, W. Huber, Electron spin resonance spectroscopy of organic radicals. Wiley-VCH (2003).

18. Neese, F. The ORCA program system. *WIREs Comput. Mol. Sci.* **2**, 73–78 (2012).

19. S. Grimme, J. Antony, S. Ehrlich, H. Krieg, *J. Chem. Phys.* **132**, 154104 (2010).

20. Weigend, F. & Ahlrichs, R. Balanced basis sets of split valence, triple zeta valence and quadruple zeta valence quality for H to Rn: Design and assessment of accuracy. *Phys. Chem. Chem. Phys.* **7**, 3297–3305 (2005).

21. Aquilante, F. *et al.* Molcas 8: New capabilities for multiconfigurational quantum chemical calculations across the periodic table. *J. Comput. Chem.* **37**, 506–541 (2016).

22. Roos, B. O., Lindh, R., Malmqvist, P.-Å., Veryazov, V. & Widmark, P.-O. Main Group Atoms and Dimers Studied with a New Relativistic ANO Basis Set. *J. Phys. Chem. A* **108**, 2851–2858 (2004).

23. Roos, B. O., Lindh, R., Malmqvist, P.-Å., Veryazov, V. & Widmark, P.-O. New Relativistic ANO Basis Sets for Transition Metal Atoms. *J. Phys. Chem. A* **109**, 6575–6579 (2005).
